# *COX4-like*, a Nuclear-Encoded Mitochondrial Gene Duplicate, Is Essential for Male Fertility in *Drosophila melanogaster*

**DOI:** 10.3390/genes13030424

**Published:** 2022-02-25

**Authors:** Mohammadmehdi Eslamieh, Ayda Mirsalehi, Dragomira N. Markova, Esther Betrán

**Affiliations:** Department of Biology, The University of Texas at Arlington, Arlington, TX 76019, USA; mmeslamieh@gmail.com (M.E.); ayda.mirsalehi@uta.edu (A.M.); markova.mira@gmail.com (D.N.M.)

**Keywords:** nuclear-encoded mitochondrial gene, gene duplication, CRISPR knockout, *COX4L*, spermatogenesis, *Drosophila melanogaster*

## Abstract

Recent studies on nuclear-encoded mitochondrial genes (N-mt genes) in *Drosophila melanogaster* have shown a unique pattern of expression for newly duplicated N-mt genes, with many duplicates having a testis-biased expression and playing an essential role in spermatogenesis. In this study, we investigated a newly duplicated N-mt gene—i.e., *Cytochrome c oxidase 4-like* (*COX4L*)—in order to understand its function and, consequently, the reason behind its retention in the *D. melanogaster* genome. The *COX4L* gene is a duplicate of the *Cytochrome c oxidase 4* (*COX4*) gene of OXPHOS complex IV. While the parental *COX4* gene has been found in all eukaryotes, including single-cell eukaryotes such as yeast, we show that *COX4L* is only present in the Brachycera suborder of Diptera; thus, both genes are present in all *Drosophila* species, but have significantly different patterns of expression: *COX4* is highly expressed in all tissues, while *COX4L* has a testis-specific expression. To understand the function of this new gene, we first knocked down its expression in the *D. melanogaster* germline using two different RNAi lines driven by the *bam-Gal4* driver; second, we created a knockout strain for this gene using CRISPR-Cas9 technology. Our results showed that knockdown and knockout lines of *COX4L* produce partial sterility and complete sterility in males, respectively, where a lack of sperm individualization was observed in both cases. Male infertility was prevented by driving *COX4L*-HA in the germline, but not when driving *COX4*-HA. In addition, ectopic expression of *COX4L* in the soma caused embryonic lethality, while overexpression in the germline led to a reduction in male fertility. *COX4L*-KO mitochondria show reduced membrane potential, providing a plausible explanation for the male sterility observed in these flies. This prominent loss-of-function phenotype, along with its testis-biased expression and its presence in the *Drosophila* sperm proteome, suggests that *COX4L* is a paralogous, specialized gene that is assembled in OXPHOS complex IV of male germline cells and/or sperm mitochondria.

## 1. Introduction

Mitochondria not only produce a large fraction of cellular energy, but are also involved in a diverse set of cellular functions, such as metabolism [1], immune regulation [2] and cell death [3]. Mitochondria are organelles in eukaryotic cells with their own DNA (mtDNA), within which, during its approximately 1.5 billion years of evolution, the mitochondrial genome has experienced many changes. Some ancestral proteobacterial genes have been lost, while others that encode mitochondrial proteins have been transferred to the nucleus (i.e., nuclear-encoded mitochondrial genes, or N-mt genes). Consequently, few genes have remained in the mitochondrial genome, which in most metazoans consists of 13 protein-coding genes, 2 rRNA genes, and 22 tRNA genes [4]. Because of this severe reduction in gene content, mitochondria import most of their proteins (N-mt proteins) from the cytoplasm. Therefore, the N-mt genes encompass genes that had mitochondrial functions and relocated to the nucleus or evolved to gain mitochondrial functions; they are transcribed in the nucleus, translated in the cytoplasm and, ultimately, the resulting proteins enter the mitochondria via five distinct transport pathways [5]. Because the mitochondrial proteins are encoded by two different genomes, the successful interactions between N-mt proteins and the 13 proteins encoded by the mitochondrial DNA are critical for all mitochondrial functions [6,7]. These are all protein–protein interactions between subunits encoded by these two genomes in most of the mitochondrial OXPHOS complexes (complexes I and III–V), which are essential for adenosine triphosphate (ATP) production.

In *D. melanogaster* there are 786 N-mt protein-coding genes (http://www.biomart.org, accessed on 28 June 2019; Ensemble Gene96, BDGP 6.22; [8]). It is estimated that 24% of the N-mt genes belong to gene families, and many (54%) of the duplicated genes have acquired tissue-specific expression [9]. Intriguingly, all N-mt duplicated genes in *D. melanogaster* with tissue-specific expression are testis-specific genes [9]. The unique expression pattern of these new genes is unexpected, given that the testis is not the only tissue with high energy demands. New N-mt genes are duplicates of the genes that encode for different mitochondrial compartments, such as OXPHOS complexes, the TCA cycle, mitochondrial membranes, redox activity, and protein folding [9,10]. These genes are enriched for energy-related functions, and considered or even observed to replace the parental gene during spermatogenesis or in mature sperm [11].

The unique expression pattern and high enrichment for energy-related functions of these newly duplicated genes has led to several nonexclusive hypotheses about their retention in the fly genome; they might have evolved in response to male mtDNA-harming mutations, to resolve intralocus sexual conflict at the parental gene, to partition the pattern of expression, or to have more of the same protein [12,13]. Some of the proteins encoded by these new genes are present in the *Drosophila* sperm proteome (DSP) [11]. Specifically, 61% (17/28) of the N-mt duplicated genes with testis-biased expression are present in the DSP, while their parental counterparts are not [10]. Some N-mt duplicated genes have been found to be essential during spermatogenesis [14,15,16,17] or under positive selection, indicating persistent selection on their functions [18]. Some studies have shown that the parental gene is capable of rescuing the phenotype of the duplicated gene, indicating that the two genes might have a similar function [19], but fails to rescue it in some other instances, indicating that the two genes have different functions [20,21]. These studies suggest that many of these duplicated genes play important/specialized roles in spermatogenesis and/or mature sperm functions.

In *D. melanogaster* there are more N-mt duplicated genes with testis-specific expression for OXPHOS complex subunits than for any other mitochondrial compartment (12/39; 31%) [22]. Cytochrome c oxidase (mitochondrial respiratory complex IV) is the last complex in the mitochondrial electron transport chain, and also one of the major regulation sites for oxidative phosphorylation [23]. The 13 subunits that form this complex are encoded by both the mitochondrial and the nuclear genomes. The three biggest subunits (COXI, COXII, and COXIII) are homologous to their corresponding subunits in prokaryotes [24], and are encoded by the mtDNA. The remaining 10 subunits, including some other cytochrome-c-oxidase-specific regulatory proteins, are encoded by the nuclear genome (N-mt genes that only exist in eukaryotes) [25,26,27]. These N-mt subunits have been proposed to modify the catalytic activity and stability of the holoprotein at complex IV [23,28]. *COX4* (*CG10664*) in *Drosophila* is one of the complex IV N-mt genes that has been duplicated through RNA-mediated duplication. The new copy, called *COX4L* (*CG10396*), is believed to still encode a subunit in that complex. *COX4L* is an old duplicate (at least 63 My old; time of *Drosophila* genus diversification), and it is present in all *Drosophila* species [29]. *COX4* is a highly conserved gene found in all eukaryotes, but so far it has not been found in bacteria that also lack this subunit. In *D. melanogaster*, *COX4* has a high expression in every tissue, and is considered to be a non-tissue-biased/housekeeping gene (sex biased ratio or SBR = 0.58) [30]. *COX4L*, however, is highly expressed in the testis, and is considered to be a male-biased gene (SBR = 9). Since *COX4L* is present in the sperm proteome but the parental gene is not [11], *COX4L* might replace the parental gene function in sperm mitochondria. Transcriptional studies of testes using GeneChip and RNA-Seq analyses have shown that the maximum expression of *COX4L* occurs at the proximal region of the testis, where the expression is significantly higher in meiosis than post-meiosis or mitosis during spermatogenesis [31,32]. The parental gene, *COX4*, has been the subject of multiple studies in a variety of organisms. For example, a reduction in COX activity, impaired ATP production, and elevated ROS production have been reported in human patients with disabling mutations in *COX4I1*—the ortholog of *Drosophila COX4* [33]. Similarly, the knockdown of *COX4* expression in *D. melanogaster* showed a reduction in the rate of mitochondrial respiration, walking speed when driven with an *arm-Gal4* driver (which drives ubiquitous expression in embryos and larvae), and complete lethality with either *da-Gal4* or *tub-Gal4* drivers (which drive ubiquitous expression in all developmental stages) [34]. 

Here, we study the phylogenetic distribution of *COX4L* and its function in *D. melanogaster* in order to understand the evolutionary pressures that have led to the retention of this duplicated gene after its origination. Results from both knocking down *COX4L* expression in the germline and knocking out the gene from the genome suggest that this gene is essential for male fertility. This prominent phenotype, along with having energy-related functions, testis-biased expression, and a presence in the *Drosophila* sperm proteome database—in which *COX4* is absent—suggests that males might use different mitochondria in their germline, and that selection might favor different, higher energy-producing mitochondria in the male germline and/or mature sperm than in the female germline and the soma. Through phylogenetic analyses, we also show that *COX4L* is older than previously thought, and is present in the Brachycera suborder of Diptera.

## 2. Materials and Methods

### 2.1. COX4L RNAi and Viability in Addition to Fertility Tests in Knockdown Flies

Flies were raised on a standard cornmeal/malt medium at room temperature (25 °C). All crosses were performed at room temperature, except for crosses set up to express a UAS transgene under a Gal4 driver for RNAi, which were run at either 25 °C or 27 °C. These temperatures were chosen because although it has been previously reported that the optimal temperature for Gal4-UAS function is 29 °C [35], this temperature has been shown to be detrimental to spermatogenesis, thus affecting both viability and fertility results [36]. Transgenic flies with UAS-RNAi constructs (i.e., RNAi lines) of KK and GD libraries were obtained from the Vienna *Drosophila* Resource Center (VDRC) [37]. The GD library insertions are P-element-based transgenes with random insertion sites, whereas the KK library contains phiC31-based transgenes with a single, defined insertion site [37]. Information for all of the lines used in this study is provided in Supplementary Table S1. The *Actin5c-Gal4* driver (a ubiquitous driver) was crossed to the RNAi lines to study the effects of knockdown in every tissue with a viability test. This driver was obtained from the Bloomington *Drosophila* Stock Center (Stock #4414). The *bam-Gal4* driver (a germline driver) was crossed with the RNAi lines to study the effects of knockdown in male and female germlines with a fertility test [38], and was obtained from the Michael Buszczak Laboratory at UT Southwestern Medical Center. The original strains that were used to make the KK and GD libraries were obtained from the VDRC and used as knockdown controls; these are the isogenic strain *w^1118^* (VDRC ID 60000) for the GD line and the *y,w[1118];P{attP,y[+],w[3`]}* strain (VDRC ID 60100) for the KK line (see Supplementary Table S1). Reciprocal crosses with at least three replicates were performed for all experiments. For the viability test, virgin males and females were collected from both strains and kept for three days to make sure that they were mature, and then two males were crossed with three females. On day five, flies were dumped out from the vials, and then the number of offspring was counted on day 15. All viability crosses were made at two different temperatures: 25 °C and 27 °C. Since the *Actin5c-Gal4* driver is against a balancer chromosome (Supplementary Table S1), viability was scored for the individuals not carrying the balancer chromosome. For the fertility test, one virgin male and two virgin females, with either the male or the female being an individual where RNAi was driven, were kept in a vial for five days, and then the number of progeny was counted on day 15. All fertility crosses were performed at two different temperatures: 25 °C and 27 °C. For both tests, data were analyzed with the R stats package (http://www.r-project.org, accessed on 20 July 2019) [39]. 

### 2.2. Generating COX4 and COX4L Knockouts and Performing Viability as Well as Fertility Tests

We used CRISPR-Cas9 technology to generate *COX4* and *COX4L* knockout flies. Two guide RNAs (gRNAs) were designed using the online platform http://tools.flycrispr.molbio.wisc.edu/targetFinder (accessed on 10 July 2019) [40], and synthesized by IDT, Inc. Then, each gRNA was annealed, phosphorylated, and ligated into the *Bbs*I sites of a pU6-*Bbs*I-chiRNA plasmid (Addgene # 45946), separately producing two plasmids to express the guide RNAs in the germline upon embryo [41]. In addition, two homologous arms were designed with the same tool to be assembled in the donor vector cloned into a pHD-DsRed-attP plasmid flanking the eye driven DsRed cassette, designed to replace *COX4* and *COX4L* (Addgene # 80898) [40]. An NEBuilder HiFi DNA Assembly Kit (NEB, Inc.) was used to assemble the homologous arms flanking the DsRed cassette. The two gRNA plasmids and the donor vector were co-injected into pre-blastoderm embryos of nos-Cas9 attp2 by Rainbow Transgenic Flies, Inc. (Camarillo, CA). The final concentration of injected plasmids for the pHD-DsRed-attP donor vector and each of the pU6-*Bbs*I-chiRNAs containing the guide RNAs was 250 ng/μL and 20 ng/μL, respectively. The gRNAs and homologous arm sequences are provided in Supplementary Tables S2 and S3. Flies were collected from injected embryos and crossed with *w^1118^* flies. The progeny of these crosses were screened for fluorescent glowing eyes, intended to confirm the replacement of the desired gene by the eye-driven DsRed gene. The mutant allele was fixed using balancer chromosomes. The absence of the genes was tested for in the homozygote mutant individuals via PCR and sequencing. Two primers inside the *COX4* and *COX4L* genes were designed for this purpose (Supplementary Tables S2 and S3). Our results confirmed that, in the case of *COX4L*, the gene was excised from the genome (Supplementary Figure S1). In the case of *COX4*, PCR and sequencing analysis of the heterozygote mutant line showed the presence of the entire *COX4* gene adjacent to the DsRed reporter, highlighting the essential function of this gene in *D. melanogaster*, which will make the generation of even the heterozygote null mutant of this gene impossible.

A viability test was performed for *COX4L*-KO flies, wherein the heterozygote virgin males and females (*COX4L*-KO/CyO) were allowed to mate for 5 days at 25 °C. Flies were discarded from the vial at day five. The number of homozygous offspring was counted on day 15 and made relative to 1/3, i.e., the portion expected of *COX4L*-KO/*COX4L*-KO flies without any viability effect. For the fertility test of *COX4L*-KO, one homozygote virgin male and two control virgin females were allowed to mate for five days at 25 °C, and then the progeny were counted on day 15. T-tests were performed with the R stats package (http://www.r-project.org, accessed on 20 July 2019) [39].

### 2.3. Rescue of the COX4L-KO with COX4L and COX4 Transgenes

FlyORF stocks of *COX4L* (fly line ID: F002652) and *COX4* (fly line ID: F005047) were obtained from the Zurich ORFeome Project Center and used to rescue the *COX4L*-KO phenotype. FlyORF stocks were created using the site-specific ΦC31 integrase to insert the transgenes (ORFs under UAS in the genome) into an identical integration site on the right arm of the third chromosome (attP-86Fb) [42]. These UAS-ORFs are under the UAS regulatory region, and can be expressed in vivo using the Gal4-UAS system. UAS-ORF lines are a valuable stock that can be used either for the ectopic expression, overexpression, or expression of a gene in a knockout mutant to rescue the loss-of-function effects. We drove *COX4L* UAS-ORF with both *bam*- and *nos*-*Gal4* drivers as well as *COX4* UAS-ORF with bam-Gal4 to rescue the *COX4L*-KO infertility phenotype. 

### 2.4. Ectopic Expression of COX4L and Overexpression of COX4 and COX4L 

The *COX4* and *COX4L* UAS-ORF lines were used to study the effect of the ectopic expression of COX4L in the soma, the overexpression of COX4L in the germline, and the overexpression of COX4 in the soma and germline. The *Actin5c-Gal4* ubiquitous driver was used for driving these genes in the soma, and the *bam-Gal4* driver was used for overexpression in the germline. Three replicates were performed at 25 °C for all crosses. To pinpoint the developmental stage affected by the lethality effect of *COX4L* overexpression, *COX4L* UAS-ORF flies were crossed with *Actin5c-Gal4* flies and placed in chambers with plates containing agar mixed with molasses and yeast paste in the middle of the plate. The embryos were collected in agar plates every 3 h, and the first-instar larvae were collected every 24 h. All embryos and larvae were transferred to vials containing media (three vials per sample). Vials were kept at 25 °C, and adult flies were counted after 15 days. 

### 2.5. MitoTracker Staining in Testes

The testes of one-day-old virgin males were dissected in 1X PBS within 20 min, fixed on slides, and stained following a formaldehyde fixation protocol [43]. Briefly, the testes were fixed in 4% formaldehyde in PBS plus 0.1% Triton X-100 (PBS-T) for 7 min, and then washed twice in PBS for 5 min at room temperature. Slides with fixed testes were immersed in PBS-T for 30 min at room temperature to permeabilize cell membranes, and then washed twice in PBS. MitoTracker™ Deep Red FM at a concentration of 500 nM (Cat # M22426, Invitrogen, Thermo Fisher Scientific) was used to stain mitochondria. The slides were washed twice in 1% PBS and then NucBlue™ Fixed Cell ReadyProbes™ Reagent (DAPI; catalog # R37606; Invitrogen, Thermo Fisher Scientific) was used to stain the nuclear DNA. The confocal microscope at UT Arlington (Nikon Eclipse Ti2 laser scanning confocal microscope) was used for imaging. NIS-Elements imaging software (version 5.20.00) was used for image visualization. In the testis of *D. melanogaster*, mitochondria run through the longitudinal axis of the tail of an elongating spermatid [44]; COX4L was expected to be present in the sperm based on *Drosophila* sperm proteome data [11]. We compared mitochondria in the sperm bundles, consisting of 64 elongated spermatids, before the individualization step, which was disrupted in the mutant.

The fluorescence observed at 60× magnification on the sperm bundles was quantified by the NIS-Elements imaging software by choosing 15 random, equally sized regions of interest, and the values were compared between *COX4L*-KO and the line of control (*w^1118^*). A t-test was performed to evaluate whether the observed difference in glowing was statistically significant between these two lines (Supplementary Figure S2).

### 2.6. Computational Analyses

Previous analyses of N-mt duplicated genes have revealed that *COX4* and *COX4L* are in the same gene family [9,10], as they have more than 50% identity at the protein level (51.4% in *D. melanogaster*). To explore whether both genes have the same function, we analyzed both genes for the presence of a mitochondrial localization signal (MLS). We used an online webserver, MitoFates [45], which analyzes the 100 amino acids from the N-terminus of any given peptide and reports the probability of a mitochondrial pre-sequence and a cleavable localization signal with their position. To compare the protein structure of both genes, domains of both proteins were predicted by the Conserved Domain Database (CDD) [46]. STRING v.11.5 (https://string-db.org, accessed on 9 January 2022) was used to predict protein–protein interactions for both *COX4* and *COX4L* in *D. melanogaster*. In addition, we evaluated the predicted protein–protein partners by calculating the evolutionary rate covariation (ERC) using the ERC Analysis web server from Pittsburg University (https://csb.pitt.edu/erc_analysis, accessed on 14 December 2021; [47,48,49]). The ERC analyses were performed using the top genes search. In these analyses, *COX4* and *COX4L* were compared to testis-enriched N-mt gene duplicates involved in OXPHOS complexes by considering their presence in the *Drosophila* sperm proteome (DmSP-II) [11]. The highest statistically significant ERC values were retrieved (Supplementary Table S4).

### 2.7. Phylogenetic Analyses

The *COX4* and *COX4L* sequences were retrieved from the NCBI database (Supplementary Material S1). Protein sequences were aligned with MUSCLE [50]. Maximum likelihood gene trees [51,52] were constructed using the BlOSUM62 substitution model with 100 bootstrap branch support in PhyML, implemented in Geneious software. We used FigTree (Version 1.4.4) (http://tree.bio.ed.ac.uk/software/figtree, accessed on 30 April 2021) to visualize all protein phylogenies. 

Coding sequences of *COX4* and *COX4L* were aligned following protein alignments using Geneious software, and the ratio of nonsynonymous to synonymous substitution per site (*d*N/*d*S = *ω*) was estimated using the CODEML algorithm [53] implemented in EasyCodeML [54]. A branch model was used with a null model, assuming that each respective group of sequences is evolving at the same rate (one-ratio model), in addition to an alternative model in which the *d*N/*d*S ratio was fixed to *d*N/*d*S = 1. We also performed a two-ratio branch model analysis to test whether the parental and the retroduplicate genes evolved under different evolutionary constraints. A two-ratio branch model can be used to test whether there are significant *ω* differences between branches of the tree by assuming that specific branches can have an *ω* that differs from the rest of the tree [53,54,55]. In both approaches, a likelihood ratio test (LRT) [56] was conducted to perform pairwise comparisons of both models for each set of parental and duplicated genes. To be considered significant, a *p*-value must be 0.05 or lower in the LRTs.

## 3. Results

### 3.1. COX4L Is a Well-Conserved Sperm Protein Differentiated from COX4

**Figure 1:**
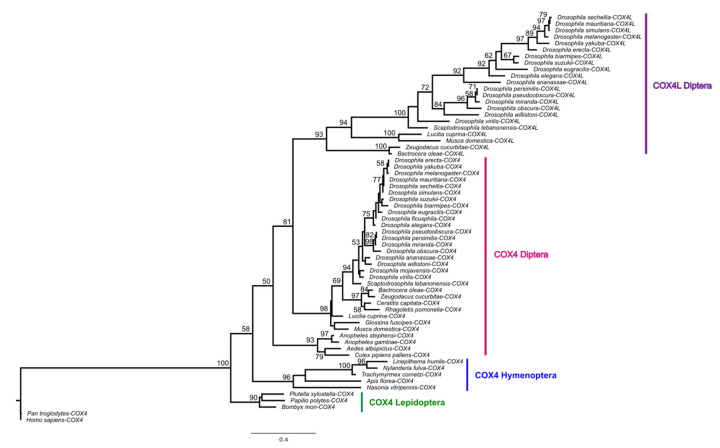
Maximum likelihood tree of *COX4* and *COX4L*, using amino acid sequences. Multiple alignments of protein sequences were performed using ClustalW [57] implemented in Geneious (Version 2020.1) [58]. The maximum likelihood phylogenetic tree was constructed using the BlOSUM62 substitution model with 100 bootstrap branch support in PhyML [52], applied in Geneious. Only bootstrap support values ≥ 50 are shown. We used FigTree (Version 1.4.4) (http://tree.bio.ed.ac.uk/software/figtree, accessed on 30 April 2021) to visualize all phylogenies.

*COX4L* is present in all *Drosophila* species, *Musca domestica*, and *Lucilia cuprina*, but not in mosquitos or more distantly related genomes (Supplementary Material S1). Thus, *COX4L* originated approximately 126 million years ago (Mya), through an RNA-mediated duplication (Supplementary Figure S1) from *COX4* (the divergence time between *Bactrocera oleae* and *D. melanogaster* is 126 Mya according to timetree.org). A phylogenetic tree using a maximum likelihood (ML) model (Figure 1) shows that the two genes cluster into two distinct clades, suggesting that they have been evolving separately since the origin of *COX4L*. Unlike *COX4*, *COX4L* is present in the DSP [11], which suggests that this gene is important for sperm function, and may have a different function than its parental counterpart. In addition, the two genes are evolving at different evolutionary rates (dN/dS*_COX4_* = 0.06967; dN/dS*_COX4L_* = 0.10732). Two-ratio branch model analyses confirm that the two genes are evolving at significantly different rates (*p* < 0.001; Supplementary Table S5), showing that each gene has different selective pressures acting on it, and that *COX4L* is evolving faster than *COX4*. In addition, the dN/dS ratio for both genes is significantly < 1 (*p* < 0.05), indicating that purifying selection is the main evolutionary force acting on both genes (Supplementary Table S5).

According to the conserved domain analysis (Supplementary Figure S3), both proteins have seven polypeptide binding sites (subunit IV/I, subunit IV/II, subunit IV/IIIb, subunit IV/Va, subunit IV/Vb, subunit IV/VIc and, subunit IV/VIIb ), with one chemical binding site (putative ATP/ADP binding site). 

A mitochondrial localization signal analysis (see Materials and Methods) predicts that both genes have a probability of over 0.9 of being imported into the mitochondria. *COX4* has one cleavage site (mitochondrial processing peptidase, or MPP, cleavage site), which is important for cleaving off pre-sequences once the protein is inside the matrix [59]. *COX4L*, on the other hand, appears to have an extra cleavage site, i.e., an intermediate cleaving peptidase of 55 kDa (Icp55), suggested to be important for protein stability within mitochondria by cleaving one amino acid from the MPP-generated intermediate N-terminus [60]. A physical protein interaction analysis using STRING v.11.5—an online biological interaction repository—is shown in Supplementary Table S6. Both COX4 and COX4L interact with many common OXPHOS complex subunits. 

The evolutionary rate covariance (ERC) can be measured across a phylogeny to find genes that directly interact and coevolve (i.e., genes that have similar evolutionary histories). Typically, a high ERC value between two genes suggests that they might be working in a common pathway or have related functions [47,48]. Therefore, the ERC value can be used to discover previously unknown functional connections between genes [49]. An ERC analysis of *COX4L* with other OXPHOS N-mt duplicated genes shows that *COX4L* has higher ERC values with other N-mt testis-enriched duplicated genes present in DmSP than its parental gene (0.3575 ERC average by query *COX4L* vs. 0.1897 ERC average by query *COX4*; Supplementary Table S4). This is the expected trend, given that only COX4L is found in the DmSP, and points to new DmSP N-mt genes working together in a specialized role in sperm. Single-cell RNA-Seq data support this interpretation [61], whereby *COX4* expression is shown to be high at the beginning of spermatogenesis and gradually decreases to being very low toward the late primary spermatocytes. On the other hand, *COX4L* expression starts very low in the spermatogonia cells, and increases to being very high in the late primary spermatocytes (Supplementary Table S7), indicative of different needs for each gene in different cell types, and pointing to different functions. Altogether, our data show that *COX4L* is a well-conserved duplicated sperm protein within the Brachycera suborder, which is also likely functionally differentiated from its parent, *COX4*. Further support for the functional differentiation between these proteins is presented below.

### 3.2. Knockdown of COX4L 

Two different strains from two RNAi libraries (KK and GD) were used to knock down the expression of *COX4L* in the soma and germline. To study the effect of this gene on viability these UAS lines were crossed with the *Actin5c-Gal4* line, and the progeny were counted (see Materials and Methods for more details). The results between different UAS libraries and temperatures (25 °C and 27 °C) were consistent with one another, and no significant differences were observed between UAS-Gal4 crosses and controls (*p* > 0.05 in all t-test comparisons; Supplementary Table S8) for any of the temperatures we used. These results suggest that *COX4L* is not required for viability.

**Figure 2:**
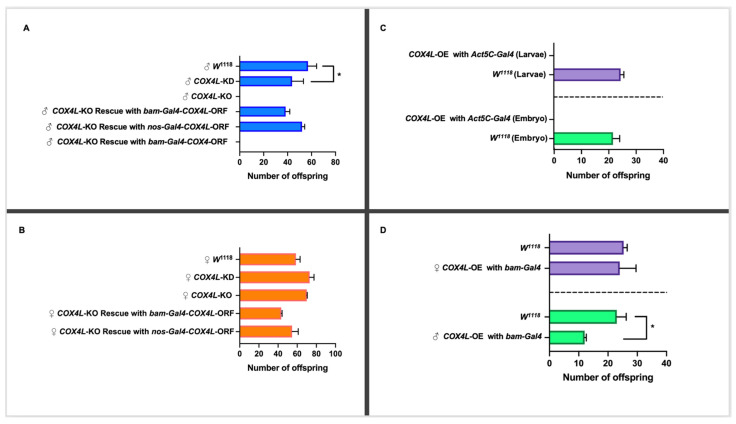
(**A**) Fertility study of *COX4L* in males. The knockdown of *COX4L* with *bam-Gal4* was semi-fertile, while the *COX4*-KO males were completely sterile. The *COX4L*-KO fertility phenotype was completely rescued by *bam-Gal4-COX4L*-ORF and *nos-Gal4-COX4L*-ORF (*p*-Value = 0.73 (*nos-Gal4*) and *p*-Value = 0.63 (*bam-Gal4*), respectively), but not with *bam-Gal4-COX4*-ORF. (**B**) Fertility study of *COX4L* in females. The overexpression of *COX4L* in the soma did not show any viability effect in females. (**C**) The overexpression of *COX4L* with *Act5C-Gal4* in the soma showed complete lethality at the embryo stage. (**D**) The overexpression of *COX4L* with *bam-Gal4* in the germline does not affect female fertility; it does, however, cause significant fertility reduction in males. (*) Means significantly different from the line of control (*w^1118^*) in a *t*-test (*p* < 0.05).

To study the effect of *COX4L* on fertility, the RNAi lines (GD and KK libraries) were driven with *bam-Gal4* (which drives expression in male and female germline cells). This Gal4 driver is expressed in the germarium, cyst cells, spermatogonia, cystoblasts, and cystocytes [38]. The UAS lines were crossed with the *bam-Gal4* line, and the virgin knockdown males and females were collected and crossed with virgin females and male *w^111^*^8^ flies, respectively. The progeny of the crosses were counted for the experimental and control crosses (see Materials and Methods). The knockdown of *COX4L* in the germline causes semi-sterility in males (*p*-Value = 0.043; Figure 2A and Supplementary Table S9). However, the knockdown of *COX4L* causes an increase in female fertility. The fertility crosses were performed at 25 °C and 27 °C, but not at 29 °C, because this temperature has been shown to have a detrimental effect on male fertility [36]. Our results were consistent between the two knockdown libraries, across both temperatures (25 °C and 27 °C), and between reciprocal crosses (32% reduction in male fertility and 26% increase in female fertility; Figure 2B and Supplementary Table S9). The results of *COX4L* knockdowns in the soma and germline were consistent with the unique expression pattern of this gene, in which only male infertility was expected.

### 3.3. Knockout of COX4L Results in Male Sterility

To confirm the results from the knockdown experiments, and to study the function of *COX4L* in more detail, we generated a *COX4L*-null mutant. Taking advantage of CRISPR-Cas9 technology, the entire coding region of *COX4L* was removed from the genome (*COX4L*-KO mutants), and this region was replaced by an eye-driven DsRed gene (see Materials and Methods for more details). Results are shown for one line only. No significant change in viability was observed between homozygote *COX4L*-KO individuals and the controls (*p* > 0.05; Supplementary Table S10). However, when we performed the fertility assay on *COX4L*-KO flies, males were completely sterile (Figure 2A). This is a recessive phenotype, as *COX4L*-KO heterozygous males are fertile. Male complete infertility was observed on six independent knockout lines, i.e., we could not fix the stock despite crossing homozygote males and females and strains had to be maintained with the balancer chromosome segregating. 

### 3.4. Male Sterility in COX4L-KO Mutants Is Due to an Individualization Defect

**Figure 3:**
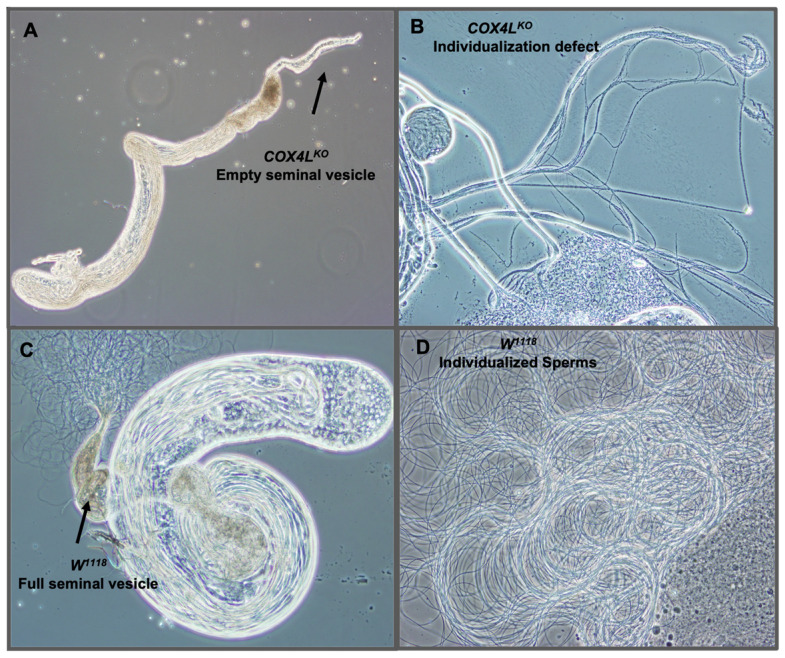
Dissected testes of *COX4L*-KO: (**A**) Dissected testes of *COX4L*-KO with an empty seminal vesicle are shown. (**B**) Sperm bundle with individualization defect. (**C**) *w^1118^* control testis with normal spermatogenesis stages and a full seminal vesicle. Additionally, mature sperms are shown moving around the raptured seminal vesicle. (**D**) Wild-type mobile individualized sperm.

We dissected the testes of homozygote knockout flies to study their sterility phenotype, and observed empty seminal vesicles and sperm bundles that failed to individualize and produce mobile sperm (Figure 3A,B). These observations suggest that a defect in the sperm individualization step occurs in *COX4L* knockouts, resulting in non-obstructive azoospermia in these flies.

**Figure 4:**
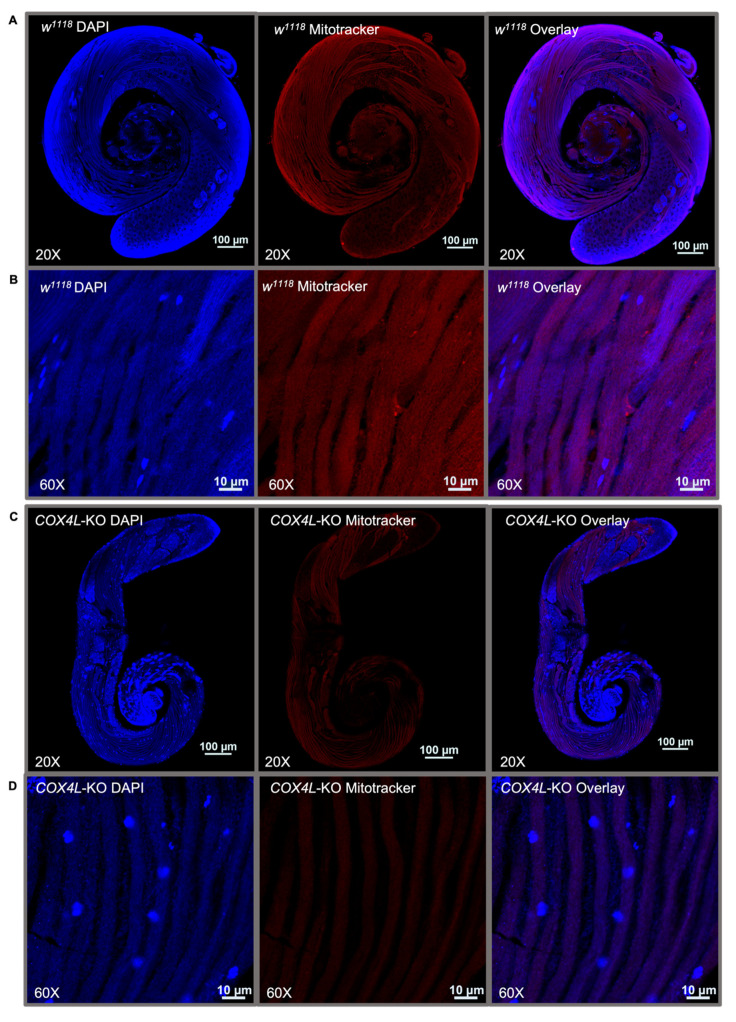
One-day-old male testes of (**A**,**B**) *w^1118^* and (**C**,**D**) *COX4L*-KO stained with MitoTracker™ Deep Red FM and DAPI. Sperm bundles stained with MitoTracker in (**B**) *COX4L*-KO show less fluorescence compared to (**D**) *w^1118^*, due to reduced mitochondrial membrane potential and/or mitochondrial morphology changes (Supplementary Figure S2).

We stained the mitochondria in the wild-type and *COX4L*-KO testes (Figure 4 and Figure S2). MitoTracker^®^ dyes passively diffuse across the plasma membrane and accumulate in active mitochondria. The quantification of fluorescence in the mitochondria of *COX4L*-KO testes after staining with MitoTracker™ Deep Red FM showed a significantly weaker fluorescence signal—on average three times less in the sperm bundles of *COX4L*-KO compared to the control (*w^1118^*) (t-test; *p* = 1.49×10^−10^; Figure 4 and Figure S2). When stained with MitoTracker, cells with reduced mitochondrial membrane potential might fluoresce less [62,63]. Therefore, we interpret this as a reduction in mitochondrial membrane potential; the change in morphology of the sperm bundles is also observable (*COX4L*-KO testes sperm bundles appear to be thinner; Supplementary Figure S2).

### 3.5. Loss-of-Function Mutation of COX4 Is Not Tolerated in Drosophila Melanogaster

In this study, two gRNAs (Supplementary Table S3) were designed to target the entire reading frame of the *COX4* gene. gRNAs were designed so that there were no mismatches between the gRNA target site and the gRNA sequence on *COX4* in the strain used for injection. The PCR screening of the resulting lines revealed that the deletion of a single copy of *COX4* is lethal; thus, the modification of this essential gene is not tolerated in *D. melanogaster*. Sequencing analyses showed that in lines expressing the eye DsRed reporter, the *COX4* DNA region was not excised out, and the gene and regulatory region were intact and immediately followed by the DsRed gene. This indicates that we recovered only transformants where the downstream gRNA was successfully used and a rearrangement occurred that incorporated the DsRed gene. Our interpretation of this result is that the loss of a single copy of *COX4* is not tolerated in *D. melanogaster*. A deficiency stock spanning *COX4* has been described (Df(2L)Exel7077; FlyBase); however, we ordered this stock and were not able to confirm that the region is deleted.

### 3.6. Rescue of the COX4L-KO Phenotype

To confirm that the lack of a *COX4L* gene is responsible for the observed infertility phenotype in *COX4L*-KO males, and whether the phenotype can be rescued by the expression of the retrogene, we drove the *COX4L* FlyORF line under the *bam-Gal4* and *nos-Gal4* drivers (Figure 2). We also drove the *COX4* FlyORF line under the *bam-Gal4* driver to understand this gene’s function (Figure 2A,B). The cross scheme is presented in Supplementary Figure S4. In all crosses, male fertility was completely rescued by driving *COX4L*, and no increased fertility effects were observed for females compared to controls. However, when *COX4* was driven, the fertility phenotype was not rescued, further validating a different function between the parental and duplicated genes (Figure 2A).

### 3.7. Overexpression of COX4L in the Soma and Germline

The *COX4L* and *COX4* FlyORF lines were also used to study the overexpression/ectopic expression of these genes in the soma and germline. The ectopic expression of *COX4L* in the soma caused complete lethality when driven with *Actin5C-Gal4*—a ubiquitous driver. In the crosses between *Act5C-Gal4*/CyO and *COX4L*-ORF, no progeny without a balancer chromosome were observed (Figure 2C, Supplementary Table S11). To determine at which stage lethality occurred, we collected and examined all embryos and larvae from the overexpression crosses. The viability effect appears to have taken place before the larval stage, as no significant difference in larval viability was observed between overexpression crosses and controls (Figure 2C). More precisely, our results show that lethality occurs during embryogenesis. The overexpression of *COX4L* with the *bam-Gal4* germline driver, in the *COX4L*-ORF x Bam/TM3 crosses, did not show any viability effect (Supplementary Table S11). However, the overexpression of *COX4L* in the germline showed a significant fertility reduction in males compared to the control group (Figure 2D, Supplementary Table S12). Interestingly, female fertility did not increase when we overexpressed *COX4L* in the germline. Overexpression of the parental gene, *COX4*, did not cause any viability or fertility effects when driven with the *Act5C-Gal4* or *bam-Gal4* drivers (Supplementary Tables S11 and S12). These results suggest that COX4L has a deleterious effect in the soma, and that its fine-tuned expression is necessary for male fertility.

## 4. Discussion

Here, we studied *COX4L*—a duplicate of *COX4*—which has testis-specific expression in *D. melanogaster*. We revealed that, in addition to *Drosophila*, this duplication is present in other flies, including *M. domestica* and *L. cuprina*. This observation suggests that *COX4L* is much older than previously estimated, but its origin does not appear to coincide with the advent of giant mitochondria along the sperm tail in these flies, as this is likely an old trait present in many insects and arthropods [44].

*COX4L* is evolving at a faster rate than *COX4*, and has overlapping but different inferred protein interactions and coevolution; however, it likely replaces *COX4* at least in the sperm, as only *COX4L* has been found in the *Drosophila* sperm proteome [11], and both genes show a high probability of targeting the mitochondria. In addition, COX4L shows an extra cleavage site known to have evolved to increase protein stability in mitochondria [60]. More importantly, knockdown of *COX4L* in the germline and its complete knockout cause partial and complete infertility in males, respectively. The partial infertility of the knockdown line could be explained by the efficiency of the RNAi or the UAS-Gal4 system, which leads to the presence of enough mRNA to show some fertility effect. The complete rescue of *COX4L*-KO with the *COX4L*-ORF line, but not with the *COX4*-ORF line, confirms that the cause of male infertility is due to the absence of the duplicate, and that the parental and duplicated genes have evolved different functions. This phenotype is consistent with the absence of COX4 in sperm, where only COX4L is present. That the functions of the two genes are different is also evident from *COX4L*’s deleterious effects when it is ectopically expressed in the soma.

An autosomal recessive mutation in the *COX4I1* gene in humans has been reported to be associated with decreased COX activity in a patient’s fibroblasts, impaired ATP production, and increased ROS production [33]. Fruit fly spermatogenesis—an energy-demanding process—might proceed without COX4L until the individualization step, but may fail afterwards because of the low ATP levels. Our results show that COX4L is important for complex IV functionality late in spermatogenesis, and that the absence of this protein might cause a morphological change in the sperm bundle tails and a decrease in the mitochondrial membrane potential, reducing ATP production. Further analyses should provide more details on those effects, and reveal whether the sperm elongation process is completed in *COX4L*-KO, as it seems to be an energy-demanding step, and the non-individualized bundles might be shorter. It is also of interest at what point COX4L replaces COX4 in the mitochondria during spermatogenesis, and precisely why.

We know there is at least the replacement of COX4 by COX4L in sperm. The fact that this duplicate has energy-related functions suggests that males might use different mitochondria in their germline, and that selection may have favored different, potentially higher energy-producing mitochondria in the male germline than in the female germline and the soma if there is a cost, e.g., ROS production and mutations [9]. The selection of specialized mitochondria has been reported previously, where a distinct germline division of mitochondrial function and structure was seen between the males and females of *Drosophila* and zebrafish [64]. Mitochondria are metabolically different in male and female gametes, with the mitochondria of female gametes (oocytes) being smaller with the suppression of DNA transcription, electron transport, and free radical production. Conversely, mitochondria of male gametes (sperm) are metabolically active, in which they transcribe mitochondrial genes for respiratory electrons, and also produce free radicals [64,65] that may cause mtDNA damage, but may not be selected against because they are not passing mitochondria to their offspring. Taken together, these results support the hypothesis that *COX4L* duplication has been retained for a role in higher/specialized energy production—at least for sperm function and fertilization.

## Data Availability

Not applicable.

## Supplementary Materials

The following are available online at https://www.mdpi.com/article/10.3390/genes13030424/s1, Figure S1: *COX4* and *COX4L* gene structures; Figure S2: *COX4L*-KO and *w^1118^* sperm bundles stained; Figure S3: COX4 and COX4L conserved domain analysis; Figure S4: Cross scheme for rescues; Table S1: Strains used; Table S2: gRNA primers and homologous arm gene blocks designed to produce the *COX4L* knockout; Table S3: gRNA primers and homologous arm gene blocks designed to produce the *COX4* knockout; Table S4: Evolutionary rate covariation values; Table S5: *d*N/*d*S ratio models and results; Table S6: Physical protein interaction analysis; Table S7: Average values of single cell expression of COX4 and COX4L; Table S8: Viability results of *COX4L* knockdown; Table S9: Fertility results of *COX4L* knockdown; Table S10: Viability results of *COX4L* knockout; Table S11: Viability results of *COX4* and *COX4L* overexpression or ectopic expression; Table S12: Fertility results of *COX4* and *COX4L* overexpression in the germline; Materials S1: Protein sequences used in the phylogeny of Figure 1. Some references are cited in the supplementary materials.
