# Supplementary material for: COX4-like, a Nuclear-Encoded Mitochondrial Gene Duplicate, Is Essential for Male Fertility in Drosophila melanogaster
Source: Genes (Basel). 2022 Feb 25;13(3):424. doi: 10.3390/genes13030424 (PMC8950493; doi:10.3390/genes13030424)
Supplement: Supplementary file 1 [file genes-13-00424-s001.zip › genes-1587006-supplementary.pdf]

Supplementary Table S1. Detailed information about the strains used in this study.

Strain name	Genotype	Stock Center
COX4L-RNAi /CG10396-RNAi (ID # 1482)	*w^{1118}; P[GD414]v1482*	VDRC
COX4L-RNAi /CG10396-RNAi (ID # 106700)	*w^{1118}; P[KK102531]v106700*	VDRC
Nos Cas9 attp2	*y,sc,v; +/+; nos-Cas9*	Rainbow Transgenic Flies, Inc.
Actin5C-Gal4 (ID # 4414)	*y[1] w[*]; P{w[+mC]=Act5C-GAL4}25FO1/CyO, y[+]*	BDSC
bam-Gal4	*y[1] w[*] P{w[+mC]=bam-GAL4:VP16}1*	BDSC
nos-Gal4 (ID # 25394)	*w[*]; Pen[D14]/CyO; P{w[+mC]=GAL4-nos.NGT}9/TM6B, Tb[+]*	BDSC
w^{1118} /GD control (ID # 60000)	*w[1118]*	VDRC
w^{1118}/KK control (ID # 60100)	*y,w[1118];P{attP,y[+],w[3`]}*	VDRC

Supplementary Table S2. gRNA primers and homologous arm gene blocks (5'-3') designed to produce the *COX4L* knockout and primers to confirm the *COX4L* knockout via PCR.

COX4L guide 1 sense	CTTCGTGTAATTATGCGCAAGCACT
COX4L guide 1 antisense	AAACAGTGCTTGCGCATAATTACAC
COX4L homologous arm 1 *Eco*RI	GGGTGTCGCCCTTCGCTGAAGCAGGTGGAATgcttttcgctggcc<br>atagctctcgtggcccttgcagccactctggttcgtgccaatatagatgataacctgccaa<br>ttaacactgagggacggattgattttgatttcggcttcgaagaggcaatttggggaaaaa<br>accgtaaaaataggaaatatctaaagaatagctcgacaatttttcacaaattacaaattt<br>aattattaaataattatttggaaagtttttaaaatttttgttttcggaattgttttatttttgtgttttttttt<br>ttcatgatatttacttttaagagattggcaaatgcttcattctctaagtagagcgagattgtctt<br>taatgtcttatattttctaaagtatagcttttttaaaattcttaagggtgggccaacaatgttatt<br>gcgatttaaaaatttttgaaaaaagtcaactagttgattcttaaactttatcaaaatttcagat<br>attgaaaactggacgtgggcaaaaaaaataattattgggcaaacagttctagatttcaa<br>aaattcgatttttccgaacccagcttctttgagctgacatgacagccatttttaaaaatgtttg<br>ttttttttttgtgacaaaaaatttgatcttcataatttttgccacgccttaaacaatttttaagaa<br>gaagtaaaattttcagactatcttagtgctcaacgaagagtgcaattcagaacttaaaaa<br>gtacatctagtttgtagataaggaaactgtcatattttttttgtattcaacaaacagactaga<br>gaaatttcattttcattcgacacgagcaacacaactgtcgaatttccggatgaagtaaaa<br>aacaaaaaattgaaaagcgagtataaaataaaatacactcaaggtacagttacgacc<br>aagtAATTCTTGCATGCTAGCGGCCGCGGACATAT
COX4L guide 2 sense	CTTCGTTCTCTCGGTAGCACCATT
COX4L guide 2 antisense	AAACAATGGTGCTACCGAGAGAAC

COX4L homologous arm 2 *Xho*I	TGCATAAGGCGCGCCTAGGCCTTCTGCAGCggtgctaccgagagaacaagtggaagtagcacatcaacacgatgattttccgtaaactatgtacagaaacgtaactagcaaaatacaattcaacagcaaagtcgcttgccatttgatcgttacgtgctgaatcgggcaaatacccaatatctatagatttttgtgtctctagctgtgtaactcgactatagcatttcctcccgtttgaaattagggtgtgtatgtaaattctcagacacaacttaatttagtgtaattttagtccacgatagatatgttaagcattgaaatcgtgtcctgtgttcctttgactagtacacgtacactgcgcgtcatcagattagcgcctccctgtatgccaccgtttcatcttatgatctgtattttccattgcacgaaaatctatcaatgttattgtttttgttcactgatattccctctctctttgagaataaaaaagaggttgagagaagaacagttatctcttttattctgctttgtgttaactttggcgcaaaattgaacacgtgttttgcatcatcagattagcgcccccagttttgaatatcgtccataatttgaaaggtaggacaacaaaattttattaaaaacaaggaatcttatagaaaaaactataattgtggcaataaccgtgtcgttaccgcggcagattgcaggtccatacttcgaattgcttccaattcccacgactcctccgccaaaattcgagaaaaaagtggtataagcgcgagcaaatcgacgattccaagggtgacttagaaagctaaacatttaaagcacaacgaactaacGCTCGAGGCTCTTCCGTCAATCGAGTTCAAG
Forward primer to confirm the *COX4L* knockout	GTACACTGCCGTCGAAATGAG
Reverse primer to confirm the *COX4L* knockout	GGGTTCATTTGCAGCTGGATG

Supplementary Table S3. gRNA primers and homologous arm gene blocks (5’-3’) designed to produce the *COX4* knockout and primers to confirm the *COX4* knock-out via PCR.

COX4 guide 1 sense	CTTCGTATATTCAATCGCTAGCAAT
COX4 guide 1 antisense	AAACATTGCTAGCGATTGAATATAC
COX4 homologous arm 1 *Eco*RI	GGGTGTCGCCCTTCGCTGAAGCAGGTGGAATgcgcacttgttaaaaatgcaaattgtcctagccacaaactgaacctgataacgaagcactaccatcccaaaaagcaaggaccccggctgcgtttccgaaaaacccaaatcttgagaaaaggaaacttcactaacatgaaatgttacagagataagacgagtgaattcatttttgtacatatagatgaaaactaaccccaagtccccccctatgcttaagtgtgaatctgaccgacggtctagactttttataccctttatcccagcccagagtcggcggatcatatgaattaaatcgcaactaatacctaactctactgtaaatgtagctataagtttttgctactaccaatttacaagatgtatagttgaaggaaaaagctttcactttagtttaattatttgcgtaattatcattaaatattgttacaaacatattgcatatttataaagcccaataaagcaaaataccattaacatatttattatgccaagcggcgcttaaacacaggaatacgcgaaagtgaatacgtaatgccccatcctcgataaaatagctaaatttccgaactccagatccctatcgaaagataatcttatgtaatacaaactcaagcagcaaactatatgatacgataaagcaaattatgtgtctctgtctaatttaaatgtgtgcatttatgagatatgacatcaaatcaaaataaaatataatatcgaacaataattgttttgttgttgtttctcttctcgcggacagactacaaaaagaatgtgaaataccaaaccaaatatttcaccatgctgcgaaataaaataaaacataatagttgcccttatttgctgtttctttattttaatatatttagatttaaataccccgccgaattccgattAATTCTTGCATGCTAGCGGCCGCGGACATAT
COX4 guide 2 sense	CTTCGCTCTTAAAACGGATCATTG

COX4 guide 2 antisense	AAACCAATGATCCGTTTTAAGAGC
COX4 homologous arm 2<br>*Xho*I	TGCATAAGGCGCGCCTAGGCCTTCTGCAGCtgatccgttttaagag<br>caatgattgttattcgatttattaacagaccttttagtgtgtagattacaaacttttcgttactaa<br>aatcgccacaggcatagccaatttaattaccacttagtgagaccttttagcactcttatgtct<br>catgttccggctgttcgatttttgaacctcatgaacaaggatctgcaaggcttccaaggca<br>tttgtgttcttggcaaattccctttcaaacatgcggccgaatcttaagatcaatggtttcagtt<br>ccggcgttatcattgctttctctttttcggtcctaaaaaggcggatttcaaaggcattgccag<br>acatataggcgaacactacttacacagctcccttttttatcacctctttcaggatctggaca<br>accgtttgaatgtccccaaataatctgtacttgtctttggtagaaatttcaccgtcaaagaat<br>aacttcagttcgttgtcagttatattcttctttcgaccccattcctccagctctgccgtctcttttg<br>gtaggcgctccgagctctgctgaaaagaagtcaggcattccttgatctcagagcattcat<br>acttgatgcagatgatgcacctacctccagttcgaaatatccctagctgtctgcttcatctttt<br>ttaaatgtcctgatctgtgtttgaaagtcttctcttctttctcctttgcaactgtcgcctttggttcg<br>gcatctcggttaaagctattgaaagcgaaaggttggtgccattcgaaagtgtatatatcct<br>tcagctcgatcgaatctgtatcttactgtttgtgccaatcgtatccatcgtatggaggcctgt<br>gcagattgtgtgctcggagatgacgctgtctttccatctgggtgtggttctggttaccGCT<br>CGAGGCTCTTCCGTCAATCGAGTTCAAG
Forward primer to confirm the *COX4* knockout	ACTACTCAACAGTGCTGTGCT
Reverse primer to confirm the *COX4* knockout	GTGTAGTCCTCGTTGTGGGA

Supplementary Table S4. Evolutionary rate covariation (ERC) values calculated using the ERC Analysis Web server from Pittsburg University (https://csb.pitt.edu/erc_analysis) [1-3]. The presence of testis-enriched N-mt gene duplicates involved in OXPHOS complexes is retrieved from the *Drosophila* sperm proteome project (DmSP-II) [4].

Duplicate Gene ID	Duplicate Gene Name	DSP	DSP Absent (Query COX4)	DSP Present (Query COX4)	DSP Absent (Query COX4L)	DSP Present (Query COX4L)
CG6485	*ND-24L*	DmSP-II	-	0.091	-	0.371
CG8102	*ND-51L2*	DmSP-I	-	-0.289	-	0.424
CG2014	*ND-20L*	X	-0.073	-	0.11	-
CG11913	*ND-49L*	DmSP-I	-	0.176	-	0.023
CG5718	*SdhAL*	DmSP-I, DmSP-II	-	0.151	-	0.554
CG14508	*Cyt-c1L*	DmSP-I, DmSP-II	-	0.285	-	0.275
CG10396	*COX4L*	DmSP-I	-	0.348	-	N/A
CG5389	*ATPsynbetaL*	DmSP-I, DmSP-II	-	0.395	-	0
CG18418	*CG18418*	X	0.496	-	0.4	-
CG4701	*CG4701*	X	0.612	-	-0.056	-
CG14740	*CG14740*	DmSP-I, DmSP-II	-	0.303	-	0.829
CG6255	*Scsα2*	DmSP-I, DmSP-II	-	0.517	-	0.215
CG33791	*CG33791*	DmSP-II	-	-0.08	-	0.527
CG33092	*P5CDh2*	X	0.553	-	0.343	-
CG11401	*Trxr-2*	X	0.59	-	0.475	-
CG9920	*BEST:GH20473*	X	-0.046	-	0.166	-
Average			0.354	0.1897	0.24	0.357

Supplementary Table S5. One-ratio branch model analysis with a null model assuming that each respective group of sequences is evolving at the same rate (one-ratio model) in addition to an alternative model in which the *d*N/*d*S ratio was fixed to *d*N/*d*S = 1, conducted for *COX4* (A) and *COX4L* (B). **C)** Two-ratio branch model analysis for the comparison of *COX4* and *COX4L* evolution under different evolutionary constraints. Significantly higher rate is shown in red.

A.

**COX4**	Branch model- One Ratio	omega (dN/dS)	0.06967
		lnL	-3785.3
		np	41
	omega =1	omega (dN/dS)	1
		lnL	-4273.55
		np	40
	*P*value:	0	

B.

**COX4L**	Branch model- One Ratio	omega (dN/dS)	0.10732
		lnL	-4755.83
		np	43
	omega =1	omega (dN/dS)	1
		lnL	-5221.51
		np	42
	*P*value:	0	

C.

	**logL; 1 rate**	**Single rate**	**logL; 2 rates**	**COX4 rate**	**COX4L rate**	**2deltaL~X2**	**d.f.**	***P***
**COX4-COX4L**	-4614.928441	0.1006	-4606.91099	0.0606	0.125	16.034902	1	0.0001

Supplementary Table S6. Physical protein interaction analysis of *COX4L* with STRING (V 11.5)

COX4 — *Cytochrome-c oxidase activity. It is involved in the biological process described with: negative regulation of neuroblast proliferation; cell proliferation; mitochondrial electron transport, cytochrome c to oxygen; mitotic cell cycle; Golgi organization (182 aa)*

Predicted Functional Partners:

Protein	Description	Neighborhood	Gene Fusion	Cooccurence	Coexpression	Experiments	Databases	Textmining	[Homology]	Score
levy	*Levy, isoform A; Enzyme regulator activity; cytochrome-c oxidase activity. It is involved in the biological process described wit...*				●	●	●	●		0.999
COX6B	*Cytochrome c oxidase subunit; This protein is one of the nuclear-coded polypeptide chains of cytochrome c oxidase, the term...*				●	●	●	●		0.999
COX5B	*Cytochrome-c oxidase activity. It is involved in the biological process described with: mitochondrial electron transport, cytoch...*				●	●	●	●		0.999
COX5A	*Cytochrome c oxidase subunit 5A, mitochondrial; This is the heme A-containing chain of cytochrome c oxidase, the terminal o...*				●	●	●	●		0.999
ATPsynG	*Hydrogen-exporting ATPase activity, phosphorylative mechanism. It is involved in the biological process described with: proto...*				●			●		0.999
cype	*Cyclope (Cype) is a cytochrome c oxidase subunit VIc homolog acting as an enhancer of dpp pathway phenotypes. Cype is in...*				●	●	●	●		0.999
UQCR-C1	*Ubiquinol-cytochrome-c reductase activity; metal ion binding; metalloendopeptidase activity. It is involved in the biological pro...*				●	●	●	●		0.995
Cyt-c1	*Cytochrome c1, isoform A; Electron transporter, transferring electrons within CoQH2-cytochrome c reductase complex activit...*				●	●	●	●		0.993
COX7A	*Cytochrome c oxidase subunit 7A, mitochondrial; This protein is one of the nuclear-coded polypeptide chains of cytochrome ...*				●	●	●	●		0.993
RFeSP	*Rieske iron-sulfur protein*				●	●	●	●		0.991

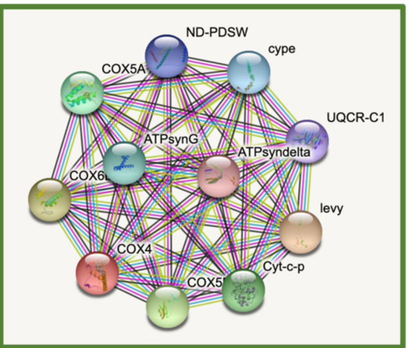

COX4L — *Cytochrome c oxidase subunit 4-like, isoform a; Cytochrome-c oxidase activity. It is involved in the biological process described with: mitochondrial electron transport, cytochrome c to oxygen (176 aa)*

Predicted Functional Partners:

Protein	Description	Neighborhood	Gene Fusion	Cooccurence	Coexpression	Experiments	Databases	Textmining	[Homology]	Score
COX5B	*Cytochrome c oxidase subunit 5b, isoform a; Cytochrome-c oxidase activity. It is involved in the biological process described...*				●	●	●	●		0.994
COX5A	*Cytochrome c oxidase subunit 5A, mitochondrial; This is the heme A-containing chain of cytochrome c oxidase, the terminal ...*				●	●	●	●		0.993
RFeSP	*Ubiquinol-cytochrome c reductase iron-sulfur subunit; Component of the ubiquinol-cytochrome c reductase complex (compl...*				●	●	●	●		0.991
levy	*Levy, isoform A; Enzyme regulator activity; cytochrome-c oxidase activity. It is involved in the biological process described wi...*				●	●	●	●		0.988
COX7C	*Cytochrome c oxidase subunit 7c, isoform a; Cytochrome-c oxidase activity. It is involved in the biological process described...*				●	●	●	●		0.988
COX6B	*Cytochrome c oxidase subunit 6b, isoform d; Cytochrome c oxidase subunit; This protein is one of the nuclear-coded polype...*				●	●	●	●		0.987
mt:CoI	*Cytochrome c oxidase subunit 1; Cytochrome c oxidase is the component of the respiratory chain that catalyzes the reductio...*				●	●	●	●		0.987
UQCR-11L	*Ubiquinol-cytochrome c reductase 11 kda subunit-like, isoform b; Cytochrome b-c1 complex subunit 6; This is a component ...*				●	●	●	●		0.985
UQCR-C2	*Ubiquinol-cytochrome c reductase core protein 2, isoform a; Ubiquinol-cytochrome-c reductase activity; metal ion binding. It i...*				●	●	●	●		0.984
UQCR-Q	*Ubiquinol-cytochrome c reductase ubiquinone-binding protein, isoform a; Ubiquinone binding; ubiquinol-cytochrome-c reduct...*				●	●	●	●		0.983

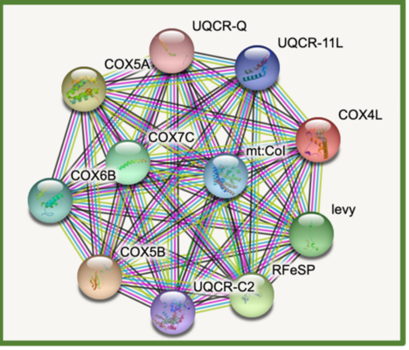

Supplementary Table S7. Average values of single cell expression of COX4 and COX4L from the spermatogonia stage to late primary spermatocytes [5].

FBgn	Gene Symbol	G (Spermatogonia)	E1° (Early Primary Spermatocytes)	M1° (Middle Primary Spermatocytes)	L1° (Late Primary Spermatocytes)
**FBgn0032833**	***COX4***	619.63	296.1673225	213.5008964	149.4138946
**FBgn0033020**	***COX4L***	167.36	1214.38135	1423.794829	1959.815878

Supplementary Table S8. Average values (± SE) of viability results of *COX4L* knockdown in the soma with the *Actin5c-Gal4* driver at 25°C and 27°C.

Crosses	Average Number of Progeny at 25 °C	Average Number of Progeny at 27 °C
♀COX4L-KK x ♂Actin5c-Gal4	91.3 ± 9.9	101.3 ± 1.7
♂COX4L-KK x ♀Actin5c-Gal4	86.7 ± 2.4	100.7 ± 6.1
♀COX4L-KK x ♂w^{1118}	80.7 ± 11.7	85.7 ± 17.8
♂COX4L-KK x ♀w^{1118}	65.0 ± 23.6	87.3 ± 15.2
♀Actin5c-Gal4 x ♂w^{1118}	78.7 ± 2.9	79.3 ± 5.0
♂Actin5c-Gal4 x ♀w^{1118}	80.0 ± 7.4	82.0 ± 7.9
♀COX4L-GD x ♂Actin5c-Gal4	80.0 ± 8.3	64.7 ± 3.5
♂COX4L-GD x ♀Actin5c-Gal4	59.3 ± 14.8	72.7 ± 2.2
♀COX4L-GD x ♂w^{1118}	85.7 ± 12.4	66.3 ± 5.7
♂COX4L-GD x ♀w^{1118}	70.7 ± 1.9	58.3 ± 9.3
♀Actin5c-Gal4 x ♂w^{1118}	67.3 ± 2.6	62.0 ± 9.3
♂Actin5c-Gal4 x ♀w^{1118}	72.7 ± 1.5	67.3 ± 6.3

Supplementary Table S9. Average values (± SE) of fertility results of *COX4L* knockdown in the germline with the *bam-Gal4* driver at 25°C and 27°C.

Crosses	Average Number of Progeny at 25 °C	Average Number of Progeny at 27 °C
♀ [♀KK x ♂Gal4](27c) x ♂W1118(25c)	69.3± 4.9	80.3± 1.2
♀[♀KK x ♂W1118] (27c) x♂W1118(25c)	57.0 ± 3.7	68.0± 1.4
♂ [♀KK x ♂Gal4](27c) x ♀W1118(25c)	44.7± 8.6	51.7± 2.9
♂[♀KK x ♂W1118] (27c) x♀W1118(25c)	72.7± 4.2	71.0± 8.1
♀[♂GD x ♀Gal4] (27c) X ♂W1118(25c)	69.0± 4.7	80.3± 1.6
♀[♂GD x ♀W1118](27c) X ♂W1118(25c)	60.3± 3.5	65.7± 2.4
♂[♂GD x ♀Gal4] (27c) X ♀W1118(25c)	47.0± 2.1	59.7± 4.1
♂[♂GD x ♀W1118] (27c) X ♀W1118(25c)	62.3± 3.3	74.0± 1.9

Supplementary Table S10. Average values (± SE) of viability results of *COX4L* knockout strain at 25°C.

Crosses	Average Number of KO/KO Progeny at 25 °C	Average Number of KO/CyO Progeny at 25 °C
♀KO/CyO x ♂KO/CyO(25c)	23.3 ± 1.8	39.7 ± 1.2
	**Average Number of +/+ Progeny at 25 °C**	**Average Number of +/CyO Progeny at 25 °C**
♀+/CyO x ♂+/CyO(25c)	24.3 ± 1.2	40.0 ± 1.0

Supplementary Table S11. Average values (± SE) of viability results of *COX4* and *COX4L* overexpression or ectopic expression in the soma and germline with the *Act-5C-Gal4* and *bam-Gal4* drivers at 25°C.

Crosses	Progeny without TM3	Progeny with TM3
COX4-ORF x *bam-Gal4*	42 ± 3.6	48.7 ± 2.3
COX4L-ORF x *bam-Gal4*	50.3 ± 3.4	47.3 ± 1.5
w^{1118} x *bam-Gal4*	48 ± 5.8	46.7 ± 2
**Crosses**	**Progeny without CyO**	**Progeny with CyO**
COX4-ORF x *Act-5C-Gal4*	47 ± 0.6	44 ± 2
COX4L-ORF x *Act-5C-Gal4*	0	48.3 ± 1.8
w^{1118} x *Act-5C-Gal4*	49.3 ± 2.8	44.3 ± 4.4

Supplementary Table S12. Average values (± SE) of fertility results of *COX4* and *COX4L* overexpression in the germline with the *bam-Gal4* driver at 25°C.

Crosses	Average Number of Progeny at 25 °C
♀ *COX4*-ORF/bam-Gal4 x ♂ w^{1118}	25 ± 1.73
♂ *COX4*-ORF/bam-Gal4 x ♀ w^{1118}	24.7 ± 3.28
♀ *COX4L*-ORF/*bam-Gal4* x ♂ w^{1118}	24 ± 5.55
♂ *COX4L*-ORF/*bam-Gal4* x ♀ w^{1118}	12 ± 0.57
♀ w^{1118}/ *bam-Gal4* x ♂ w^{1118}	25.3 ± 1.2
♂ w^{1118}/ *bam-Gal4* x ♀ w^{1118}	23.3 ± 3.18
**Crosses**	**Average Number of Progeny at 25 °C**
♀ *COX4*-ORF/ *Act-Gal4* x ♂ w^{1118}	25 ± 1.73
♂ *COX4*-ORF/ *Act-Gal4* x ♀ w^{1118}	25.3 ± 4.05
♀ *COX4L*-ORF/ *Act-Gal4* x ♂ w^{1118}	27.3 ± 2.02
♂ *COX4L*-ORF/ *Act-Gal4* x ♀ w^{1118}	29.3 ± 1.45
♀ w^{1118}/ *Act-Gal4* x ♂ w^{1118}	25.3 ± 3.84
♂ w^{1118}/ *Act-Gal4* x ♀ w^{1118}	26.3 ± 1.2

References

1. Clark, N.L.; Alani, E.; Aquadro, C.F. Evolutionary rate covariation reveals shared functionality and coexpression of genes. *Genome research* **2012**, *22*, 714-720, doi:10.1101/gr.132647.111.

2. Clark, N.L.; Alani, E.; Aquadro, C.F. Evolutionary rate covariation in meiotic proteins results from fluctuating evolutionary pressure in yeasts and mammals. *Genetics* **2013**, *193*, 529-538, doi:10.1534/genetics.112.145979.

3. Findlay, G.D.; Sitnik, J.L.; Wang, W.; Aquadro, C.F.; Clark, N.L.; Wolfner, M.F. Evolutionary rate covariation identifies new members of a protein network required for Drosophila melanogaster female post-mating responses. *PLoS genetics* **2014**, *10*, e1004108, doi:10.1371/journal.pgen.1004108.

4. Wasbrough, E.R.; Dorus, S.; Hester, S.; Howard-Murkin, J.; Lilley, K.; Wilkin, E.; Polpitiya, A.; Petritis, K.; Karr, T.L. The Drosophila melanogaster sperm proteome-II (DmSP-II). *J Proteomics* **2010**, *73*, 2171-2185, doi:10.1016/j.jprot.2010.09.002.

5. Mahadevaraju, S.; Fear, J.M.; Akeju, M.; Galletta, B.J.; Pinheiro, M.; Avelino, C.C.; Cabral-de-Mello, D.C.; Conlon, K.; Dell'Orso, S.; Demere, Z.; et al. Dynamic sex chromosome expression in Drosophila male germ cells. *Nat Commun* **2021**, *12*, 892, doi:10.1038/s41467-021-20897-y.

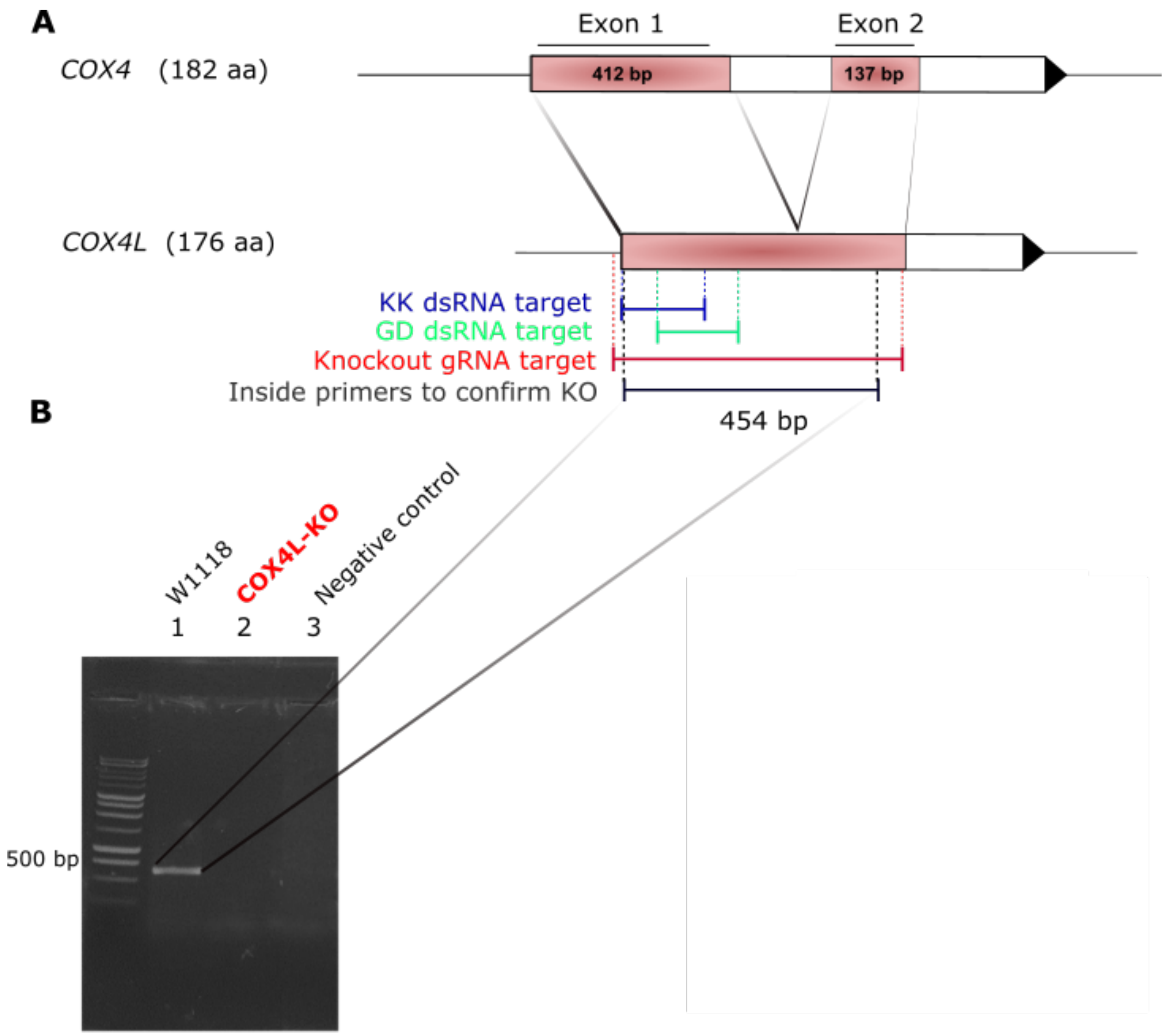

Supplementary Figure S1. (A) *COX4* and *COX4L* gene structures. *COX4L* is an RNA-mediated duplication of *COX4*, which is only six aa shorter than its parental protein. The regions of dsRNA expressed in the RNAi knockdowns using the KK and GD lines are shown. The *COX4L*-KO targeted region is shown. The location of the primers for PCR within *COX4L* is depicted. (B) The lack of a PCR amplification for *COX4L*-KO homozygotes is shown, confirming the removal of *COX4L* from the genome.

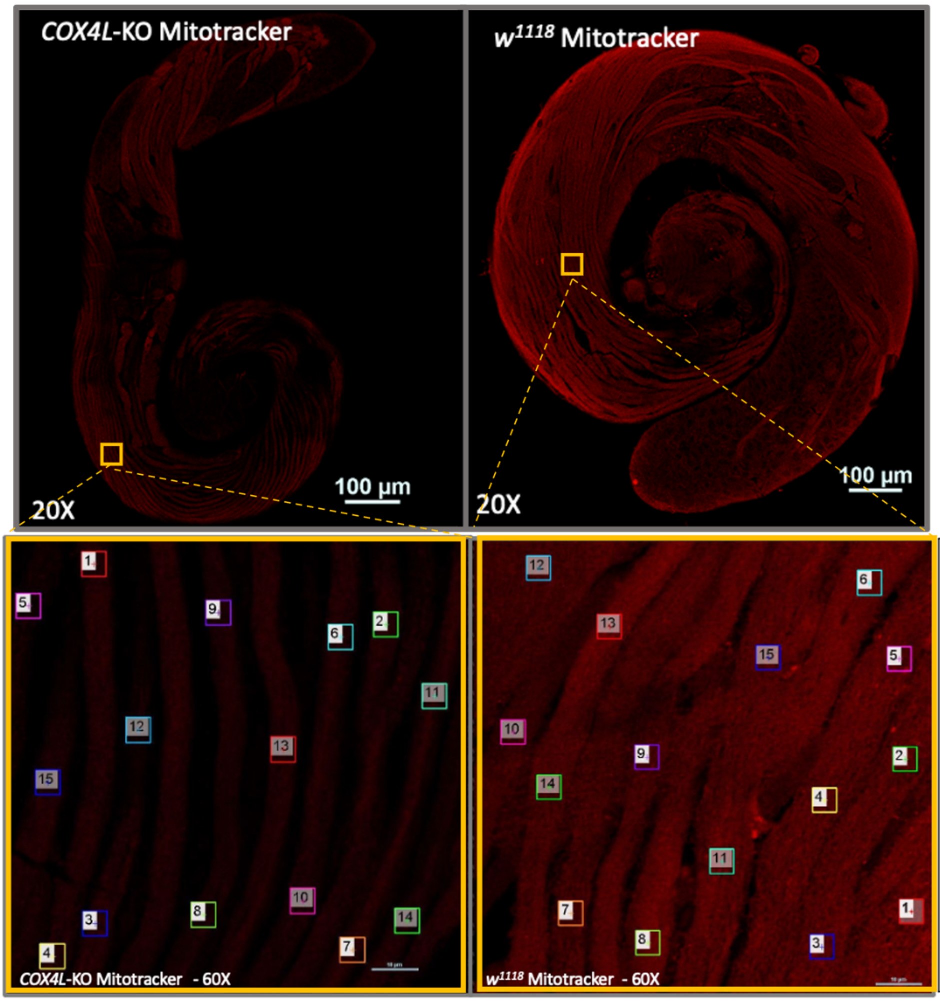

	W1118	COX4L-KO
1	1374.847	401.1494
2	1233.292	464.0166
3	1317.888	435.0584
4	1173.344	411.9317
5	1138.54	220.4361
6	1414.348	428.3314
7	955.9697	457.9955
8	1356.744	298.4862
9	976.3196	365.6724
10	744.9306	357.4796
11	1309.228	416.8616
12	950.3059	201.5548
13	1353.45	429.9167
14	1020.872	325.0104
15	1183.272	386.3094
Mean	1166.890053	373.347347
P-value	1.49139E-10	

Supplementary Figure S2. *COX4L*-KO and w^{1118} sperm bundles stained with MitoTracker™ Deep Red FM are shown. The confocal microscope at UT Arlington (Nikon Eclipse Ti2 laser scanning confocal microscope) was used for imaging. NIS-Elements imaging software (version 5.20.00) was used for image visualization. The fluorescence observed at 60X magnification on the sperm bundles was quantified by the NIS-Elements imaging software by choosing 15 random equally sized regions of interest, and the values were compared between *COX4L*-KO and the line of control (w^{1118}). A performed t-test shows statistically significant differences in the observed glowing between these two strains. We interpret this as a reduction in the mitochondria membrane potential and a change in the morphology of the sperm bundles (*COX4L-KO* testes sperm bundles appear to be thinner).

a. COX4 conserved

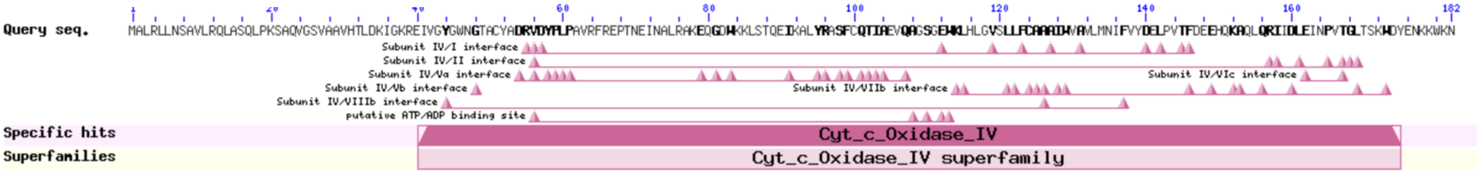

b. COX4L conserved domains

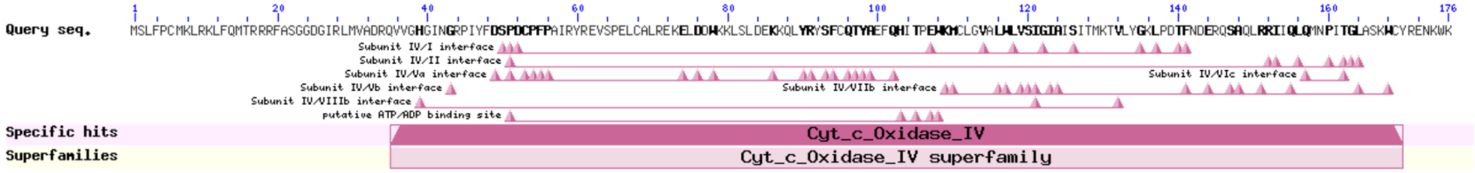

Supplementary Figure S3. COX4 (a) and COX4L (b) conserved domain analysis.

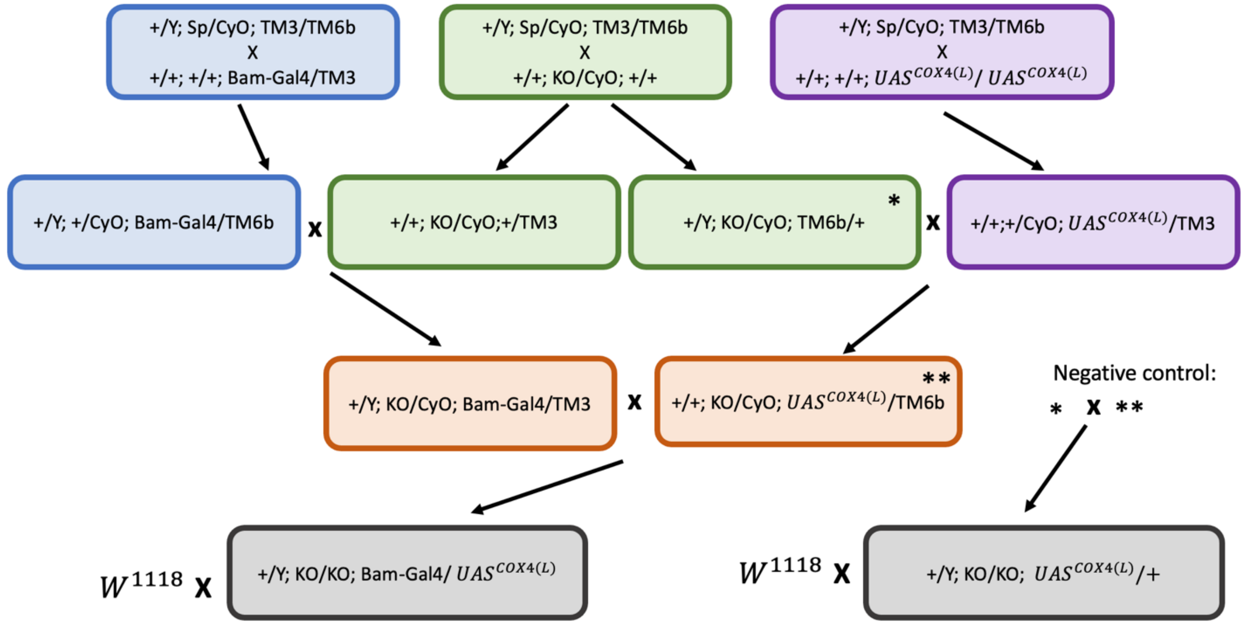

Supplementary Figure S4. Cross scheme used in this study for the rescue of the *COX4L*-KO phenotype by driving *COX4L*-ORF and *COX4*-ORF with the *bam*- and/or *nos-Gal4* drivers.

>NP_001260612.1_Drosophilamelanogaster_COX4

MALRLLNSAVLRQLASQLPKSAQVGSVAAVHTLDKIGKREIVGYGWNGTACYADRVDYPLPA

VRFREPTNEINALRAKEQGDWKKLSTQEIKALYRASFCQTIAEVQAGSGEWKLHLGVSLLFCA

AAIWVAVLMNIFVYDELPVTFDEEHQKAQLQRIIDLEINPVTGLTSKWDYENKKWKN

>XP_002039171.1_Drosophila_sechellia_COX4

MALRLINSAVLRQLASQLPKSAQVGSVAAVHTLDKIGKREIVGYGWNGTACYADRVDYPLPAV

RFREPTNEINALRAKEQGDWKKLSTQEIKALYRASFCQTIAEVQAGSGEWKLHLGIALLFSAAA

IWVAVLMNLFVYDELPVTFDEEHQKAQLQRIIDLEINPVTGLTSKWDYENKKWKN

>XP_002090761.1_Drosophila_yakuba_COX4

MALRLLNSAVLRQLASQLPKSAQVGSVAAVHTLDKIGKREIVGYGWNGTACYADRVDYPLPA

VRFREPTNEINALRAKEQGDWKKLSPQEIKALYRASFCQTIAEVQAGSGEWKLHLGVALLFTA

AAIWVAVLMNIFVYDELPVTFDEEHQKAQLQRIIDLEINPVTGLTSKWDYENKKWKN

>ABM88269.1_Drosophila_simulans_COX4

MALRLINSAVLRQLASQLPKSAQVGSVAAVHTLDKIGKREIVGYGWNGTACYADRVDYPLPAV

RFREPTNEINALRAKEQGDWKKLSTQEIKALYRASFCQTIAEVQAGSGEWKLHLGIALLFSAAA

IWVAVLMNLFVYDELPVTFDEEHQKAQLQRIIDLEMNPVTGLTSKWDYENKKWKN

>XP_015011971.1_Drosophila_erecta_COX4

MALRLLNSAVLRQLASQLPKSAQVGSVAAVHTLDKIGKREIVGYGWNGTACYADRVDYPLPA

VRFREPTNEINALRAKEQGDWKKLSPQEIKALYRASFCQTIAEVQAGSGEWKLHLGVALLFTA

AAIWVAVLMNLFVYDELPVTFDEEHQKAQLQRIIDLEINPVTGLTSKWDYENKKWKN

>AAP88318.1_Drosophila_mauritiana_COX4

MAXRLINSAVLRQLASQLPKSAQVGSVAAVHTLDKIGKREIVGYGWNGTACYADRVDYPLPAV

RFREPTNEINALRAKEQGDWKKLSTQEIKALYRASFCQTIAEVQAGSGEWKLHLGIALLFSAAA

IWVAVLMNLFVYDELPVTFDEEHQKAQLQRIIDXEINPVTGLTSKWDYENKKWKX

>XP_017081521.1_Drosophila_eugracilis_COX4

MALRLLNSAVLRQLASQLPKSAQVGSVASVHTLDKIGKREIVGYGWNGTACYADRVDYPLPA

VRFREPTNEINALRTKEQGDWKKLSQQEIKALYRASFCQTIAEVQAGTGEWKLHLGIALLFSAA

AIWVAVLMNLFVYDELPVTFDEEHQKAQLKRIIDLEINPVTGLTSKWDYENKKWKN

>XP_017056702.1_Drosophila_ficusphila_COX4

MALRLINSAVLRQLASQLPKSAQVGSVAAVHTLDKIGKREIVGYGWNGTACYADRVDYPLPAV

RFREPTNEINALRTKEQGDWKKLSKEEIKALYRASFCQTIAEVQAGTGEWKLHLGVALLFSAA

AIWVAVLMNLFVYDELPVTFDEEHQKAQLKRIIDLEMNPVTGLTSKWDYENNKWKN

>XP_016962286.1_Drosophila_biarmipes_COX4

MALRLLNSAVLRQLAAQLPKNAQVGSVAAVHTLDKIGKREIVGYGWNGTACYADRVDYPLPA

VRFREPTNEINALRTKEQGDWKKLSPEEIKALYRASFCQTIAEVQASSGEWKLHLGIAFLFSAA

AIWIAVLMNLFVYDELPVTFDEEHQKAQLQRIIDLEINPVTGLTSKWDYENKKWKN

>XP_016924725.1_Drosophila_suzukii_COX4

MALRLINSAVLRRLASQLPKNAQVGSVAAVHTLDKIGKREIVGYGWNGTACYADRVDYPLPAV
RFREPTNEINALRTKEQGDWKKLSPEEIKALYRASFCQTIAEVQAGTGEWKLHLGIALLFSAAAI
WVAILMNLFVYDELPVTFDEEHQKAQLQRIIDLEMNPVTGLTSKWDYENKKWKN

>XP_017117177.1_Drosophila_elegans_COX4
MALRLINSAVLRQLASQLPKSAQVGSVVGVHTLDKIGKREIVGYGWNGTACYADRVDYPMPA
VRFREPNNEINALRTKEQGDWKKLSREEIKALYRASFCQTIAEVQAGTGEWKLHLGVALLFSA
AAIWVAVLMNLFVYDELPVTFDEEHQKAQLKRIIDLEMNPVTGLTSKWDYENNKWKN

>XP_017154168.1_Drosophila_miranda_COX4
MALRLINHAMLRQLAAQLPRNAQVGSVASVHTLDKIGKREVVGYGWNGTACYADRVDYPMP
AVRFREPNNEINALRAKEQGDWKKLSPQEIKVLYRASFCQTIAEVQAGTGEWKQHLGVSLLFC
AGAIWIAILMNLFVYDELPVTFDEEHQKAQLKRIIDLEMNPVTGLTSKWDYENKQWKN

>XP_015036723.1_Drosophila_pseudoobscura_COX4
MALRLINHAMLRQLAAQLPRNAQVGSVASVHTLDKIGKREVVGYGWNGTACYADRVDYPMP
AVRFREPNNEINALRAKEQGDWKKLSPQEIKALYRASFCQTIAEVQAGTGEWKQHLGVSLLFC
AGAIWIAILMNLFVYDELPVTFDEEHQKAQLKRIIDLEMNPVTGLTSKWDYETKQWKN

>XP_002021764.2_Drosophila_persimilis_COX4
MPQLNNTIDSLLCNCDRMALRLINHAMLRQLAAQLPRNAQVGSVASVHTLDKIGKREVVGYG
WNGTACYADRVDYPMPAVRFREPNNEINALRAKEQGDWKKLSPQEIKALYRASFCQTIAEVQ
AGTGEWKQHLGVSLLFCAGAIWIAILMNLFVYDELPVTFDEEHQKAQLKRIIDLEMNPVTGLTS
KWDYETKQWKN

>XP_001962444.1_Drosophila_ananassae_COX4

MALRLINNAVLRQLVAQLPKNAQVGSVAGVHTLDKIGKREIVGFGWNGTACYADRVDYPLPAV
RFREANNEINALRTKEQGDWKKLSPQEIKALYRASFCQTIAEVQAGTGEWKMHLGVGLLFTAA
AIWVAVLMNLFVYDELPVTFDEEHQKAQLKRIIDLEINPVTGLTSKWDYENNKWKN

>XP_022232103.1_Drosophila_obscura_COX4

MALRLINSAVFRQLVAQLPRNAQVGSVASVHTLDKIGKREVVGYGWNGTACYADRVDYPMPA
VRFREPNNEINALRAKEQGDWKKLSPQEIKALYRASFCQTIVEVQAGSGEWKQHLGVSLLFCA
GAIWIAILMNLFVYDELPITFDDEHQKAQLKRIIDLEMNPVTGLTSNWDYEKKQWKN

>XP_023031397.1_Drosophila_willistoni_COX4

MALRLINSAVFRQLVNQLPRNAQVGSVASVHTLDKIGKREIVGYGWNGTACYADRVDYPLPAI
RFREANNEINALRAKEQGDWKKLSPQEIKALYRASFCQTIAEVQAPTGEWKMHLGVGLIFTAA
AIWIAVLMNLFVYDELPITFDEEHQKAQLKRIIDLEINPVTGLTSKWDYENNKWKN

>XP_002051184.1_Drosophila_virilis_COX4

MALRLINNALRRQLAAQLPRNAQVGSVASIHTLDKIGKREIVGFGWNGTACYADRVDYPMPAV
RFREPNNEINALRTKEQGDWKKLSPQEIKALYRASFCQTIAEVQASTGEWKMHLGVGLIFTAA
AIWIAVLMNLFVYDELPITFDEEHQKAQLKRMIDLEINPVTGLTSKWDYENNKWKN

>XP_002003948.1_Drosophila_mojavensis_COX4

MALRLINSALRRQLAAQLPHNAQVGSVASVHTLDKIGKREVVGFGWNGTACYADRADYPMPA
VRFREPNNEITALRTKEQGDWKKLSPQEIKALYRASFCQTIAEVQAGTGEWKMHLGIGLIFTAA
AIWIAVLMNLFVYDELPVTFDEEHQKAQLKRMIDLEINPVTGLTSKWDYENNKWKN

>XP_030381258.1_Scaptodrosophila_lebanonensis_COX4
MALRLINHALRRQLAAQLPRNAQVGSVASVHTLDKIGKREVVGYGWNGTASYADRVDYPLPPI
RFREPNNEINALRVKEQGDWKKLSPQEIKALYRASFCQTIAEVQAGTGEWKMHLGIGLLFTSA
AIWIAILMNLFVYDELPVTFDEEHQKAQLKRMLDLEVNPITGLSSKWDYENNKWKN

>XP_005179417.1_Musca_domestica_COX4
MALRVLNMALRRQLAAQLPRTSQVGSVASVHTLDKIGKREIVGYGWNGTACYVDRVDYPMPP
VRFREPTNEINNLREKEKGDWKKLSVEEKKALYRASFCQTLAELKAPSGEWKLHLGMGLLFA
SAAIWVAILMNLFVYDELPASFDEEHQKAQLKRMLDLEINPVAGLSSKWDYENKRWKN

>XP_023290998.1_Lucilia_cuprina_COX4
MALRVLNMALRRQLAQLPRNAQVGSVASVHTLDKIGKREIVGYGWNGTACYADRVDYPMPAI
RFREATNEINALREKEKQDWKKLSNEEKKALYRASFCQTFAEMKAGTGEWKMHLGMGLIFTT
LALWVAIFMNTYVYDEMPVTFDEEHQKAQLKRMLDLEMNPVTGISSKWDYENKRWK

>XP_014101397.1_Bactrocera_oleae_COX4
MALRGFSMSMQRQLLKQLLKNTQSGSIASVHTLDKIGNREIVGFGWNGTACYADRTDYPMPA
IRFREPTNEIKALREKEKQDWKKLSPEEIKVLYRASFCQTFAEIQAPTGEWKQHLGISFIFIGLAI
WIAVLMNLFVYDELPVTFDDEHKKAQLKRMIDLEVNPVTGLTSKWDYENKKWK

>XP_037883030.1_Glossina_fuscipes_COX4

MALRVLDMALRRQLAAQVVRCPQIGSVASVHTLDKIGKREIVGYGWNGTACYYDRADYPMPA

VRFREPTNEINNLRQKEKGDWKKMSIDEKKALYRASFCQTFAEIQAPTGEFKQHFGVGLLFTA

MAIWVAIFMNLFVYDEMPVTFDEEHKKAQLKRMIDLEMNPVTGLASKWDYQNNRWK

>XP_004534967.1_Ceratitis_capitata_COX4

MALRGISVSMQRQLFKQLLKTAQSGSVASVHTLDKIGNREIVGHGWNGTACYADRTDYPMPA

IRFREVNNEIKALREKEKQDWKKLSHEEIKALYRASFCQTFAEIQAPTGEWKQHLGMGLIFTSL

AIWIAILMNLFVYDELPVTFDEEHQKAQLRRMIDLEVNPVTGLTSKWDHENKKWK

>XP_036321723.1_Rhagoletis_pomonella_COX4

MALRGFSLPMQRQLLKQMIKTVQNGSVASVHTLDKIGNREIVGHGLNGSACYVDRTDYPMPAI

RFREPTNEIKALREKEKQDWKKLSHQEIKALYRASFCQTFAEIQAPTGEWKQHVGISLIFTGLAI

WIAILMNLFVYDELPVTFDEEHQKAQLKRMIDLEVNPVTGLASKWDYENNKWKN

>XP_011182108.1_Zeugodacus_cucurbitae_COX4

MALRGFSMSMQRQLLKQLLKNTQSGSIASIHTLDKIGNREIVGYGWNGTACYADRTDYPMPAI

RFREPNNEIKALREKEKQDWKKLSPEEVKALYRASFCQTFAEIQAPTGEWKQHLGIGFIFISMA

IWIAVLMNLYVYDEMPVTFDDEHKKAQLKRMLDLEVNPVTGLTSKWDHENKKWK

>XP_029708249.1_Aedes_albopictus_COX4

MANVNLASVVLRNALRQKMGVRFSHDMIAQKIGKREVVGHGWNGLPVYADRVDYPMPAIRF

KEVTPDVMALREKEKGDWKKLSMQEKKALYRASFCQTFSEIKYPTGEWKLSVGFGLIVLSMSL

ATMMLMKAFVYDDIPVTFDDEHQKAQLKRMLDLGVGNITGLSSKWDYENNKWK

>XP_035911113.1_Anopheles_stephensi_COX4

MANVNLASVVLRNALRTKMGTRQAHDMISQKIGKREVVGHGWNGLPVYADRVDYPMPAIRFK

ENTRDVLALREKEKGDWKKLSVQEKKALYRASFCQTFAEMKHPTGEWKACLGAALIATSMSLI

GMMLLKTFVYDPIPETFDEEHQKAQLKRMLDLNINPIHGVSSKWDYENNKWK

>XP_039447568.1_Culex_pipiens_pallens_COX4

MANVNVAQVVLRNALRTKLGSVRYASSDMMLQKIGKREVVGHGWNGMPVYADRVDFPFPAI

RFKEPTPDVLALREKEKGCWKKLSVQEKKALYRASYCQTFSEMKYPSGEWKMCLGFGLIAIS

MSITCMLLMKAFVYEKIPETFDDEHQKAQLKRMLDLGVGPVTGLSSKWDYDNNKWK

>XP_314839.4_Anopheles_gambiae_partial_COX4

LKTETMANVNLASVVLRTALRAKIGTRQSHDMITQKIGKREVVGHGWNGLPVYADRVDYPMP

AIRFKENTRDVLALREKEKGDWKKMSVQEKKALYRASFCQTFAEMKHPTGEWKACLGAALIA

ASMSLIGMMLLKAFVYEPIPETFDEEHQKAQLKRMLDLNINPIHGVSSKWDYDNNKWK

>XP_013144802.1_Papilio_polytes_COX4

MANYLLRRAIIDAIRVPVGTRASSELAKIGNREWVGYGYNGQPTYVDRPDFPLPAVRFRPDTP

DVKVLREKERGDWRKLTLEEKKALYRASFCQTFAEFQAPTGEWKGVVGWSLVFISLSLWVYM

GMKLFVYSPLPASFDEDAQKAQLKRMLDLKVNPIDGLSSKWDYENNRWK

>XP_037965467.1_Plutella_xylostella_COX4

MANLLMRRALINAIRVPAGTRASSVNTDLAKVGKREWVCYGFNGQPNYVDRPDYPMPAVRF

QPETPDIKMLREKEKGDWRKLTMEEKKALYRASFCQTFAEFQAPTGEWKGVTGWALTLASLS

VWIYFAMKIFVYSPLPETFDDEHQKAQLKRMLDLKVNPVDGLASKWDYENNRWK

>NP_001073120.1_Bombyx_mori_COX4

MANYLMRRALINAIRVPVCARAGSTGNTELAKIGDREWVGYGFNGQPNYVDRPDFPLPAIRFR

EDTPDIKALREKEKGDWRKLTLEEKKTLYRASFCQTFAEFQAPTGEWKGVVGWALVLSSLAA

WIYMAMKVFVYSPIPDSLSEERQKAQLQRMLDLKVNPIDGLASKWDYENNRWK

>XP_029177602.1_Nylanderia_fulva_COX4

MAGRLFASRLRPVIQVQRCGLMTLDRVGNRDVVGFGYNGEPTYLDRVDFPCPAIRWKENTP

DVMALKEKEKGDWKKLSIEEKKALYRASFRQTFSEIDAPTGEWKGIIGLSCIFLSAGVWLYLYF

KVFAYPELPETFSLERRLAQLDRMKKLDMNPIDGLCARK

>XP_012221014.1_Linepithema_humile_COX4

MASRLFASRLRPIIQIQKCRIMTVDRIGNRDVVGFGYNGEPVYVDRVDFPCPAIRWKENTPDV

MALREKEKGDWKKLSVEEKRALYRASFRQTFSEIDAPTGEWKGILGMCMVLISSAIWLFLYFK

TFAYPELPETFSLERRLAQLDRMKKLDMNPIDGLCARK

>XP_012340029.1_Apis_florea_COX4

MLVAISLFLELGSAKKHTMANKLLLSYLRQSTSMCVRGLSAMQFPNKIGNRDVVGNGWNGEE

AYLDRSDFPLPAIRFKANTPDIMALREKEKGDWKKLSIEEKKILYRASFRQTFSEFLAPTGEWR

GHIGIALIGVAFSLWIYIFLKIYALPPLPESFNEENRLAQLERMKLLQVNPIDGISSKN

>KYN28998.1_Trachymyrmex_cornetzi_COX4
MTMDKVGNRDVVGFGYNGEPAYLDRVDFPYPAIRWKENTPDIMALREKEKGDWKKLSIEEKK
ILYRASFRQTFSEMDAPTGEWKGILGMSLLITSAGIWLYLYFKAFAYPPLPETFSLERRLAQLDR
MKKLDMNPIDGLCARK

>NP_001165811.1_Nasonia_vitripennis_COX4
MASRALTTFLRSAAVQAQTRSVYTIQNKIGNREVVCHGMNSEPIYIDTTDFPMPAIRYKEVTPDI
QALREKEKGDWKKLSVEDKKALYRASFRQTFAEMEAPSGDWKSVIGLSLVGISISMWLFVWT
KHYVYPPLPSSLSEENQLAQLERMKLLDMQPITGLPGTKK

>AAH62437.1_Homo_sapiens_COX4
MLTTRVFSLVGKRAISTSVCVRAHESVVKSEDFSLPAYMDRRDHPLPEVAHVKHLSASQKALK
EKEKASWSSLSMDEKVELYRIKFKESFAEMNRGSNEWKTVVGGAMFFIGFTALVIMWQKHYV
YGPLPQSFDKEWVAKQTKRMLDMKVNPIQGLASKWDYEKNEWKK

>NP_001238844.1_Pan_troglodytes_COX4
MLATRVFSLVGKRAISTSVCVRAHESVVKSEDFSLPAYMDRRDHPLPEVAHVKHLSASQKALK
EKEKASWSSLSMDEKVELYRIKFKESFAEMNRGSNEWKTVVGGAMFFIGFTALVIMWQKHYV
YGPLPQSFDKEWVAKQTKRMLDMKVNPIQGLASKWDYEKNEWKK

>NP_610168.1_Drosophila_melanogaster_COX4L
MSLFPCMKLRKLFQMTRRRFASGGDGIRLMVADRQVVGHGINGRPIYFDSPDCPFPAIRYRE
VSPELCALREKELDDWKKLSLDEKKQLYRYSFCQTYAEFQHITPEWKMCLGVALWLVSIGIAIS
ITMKTVLYGKLPDTFNDERQSAQLRRIIQLQMNPITGLASKWCYRENKWK

>XP_033155789.1_Drosophila_mauritiana_COX4L

MSLFPCMKLRKLFQMTRRRFASGGDGIRLMVADRQVVGHGINGRPIYFDSPDCPFPAIRYRE
VTPELCALCEKELDDWKKLSLDEKKQLYRYSFCQTYAEFQHFTPEWKLCLGVALWLVSIGIAIS
ISMKTVLYGKLPETFNEERQSAQLRRIIQLQMNPITGIASQWCYRENKWK

>ABM88275.1_Drosophila_simulans_COX4L

MSLFPCMKLRKLFQMTRRRFASGGDGIRLMVADRQVVGHGINGRPIYFDSPDCPFPAIRYRE
VTPELCALCEKELDDWKKLSLDEKKQLYRYSFCQTYAEFQHFTPEWKLCLGVALWLVSIGIAIS
ISMKTVLYGKLPDTFSEERQSAQLRRIIQLQMNPITGIASQWCYRENKWK

>XP_002044619.1_Drosophila_sechellia_COX4L

MSLFPCMKLRTLFQMTRRRFASGGDGIRLMVADRQVVGHGINGRPIYFDSPDCPFPAIRYREV
TPELCALCEKELDDWKKLSLDEKKQLYRYSFCQTYAEFQHFTPEWKLCLGVALWLVSIGIAISI
SMKTVIYGKLPETFNEERQSAQLRRIIQLQMNPITGIASQWCYRENKWK

>EDX00443.1_Drosophila_yakuba_COX4L

MNLFPCLKLRKLYQMTRRRFASGGDSIRLMVADREVVGHGINGRPIYFDSPDCPFPAIRYQEV
NSKLCALREKELDDWKNLSLDEKKQLYRHSFCQTYAEFQHFTPEWKICLAVALWLVSVGIAISI
SMKTMLYGKLPQTFDDERQSAQLRRIIQLQMNPITGLSSKWCYQENKWK

>XP_001971288.1_Drosophila_erecta_COX4L

MSLIPCLRGRNLFQMTRRGFASGGDLIRLMVADREVVGYGINGRPIYFDSPDCPFPAIRYREV
NPELCALREKELDDWKKLSLDEKKQLYRHSFCQTYAEFQHFTPEWKLCLGVALWVVALGIGIS
ISMKTLVYRKLPDTFDDEHQSAQLRRIIQLQMNPITGISSKWCYHENKWK

>XP_016937064.1_Drosophila_suzukii_COX4L
MSALRLIPRNLSQLTTPTRMAMMGRRFASGGDGVRLLIGDREVVGYGINGRPLYFDSPDCPF
PAIRYREVTPELCAVREKELGDWKKLSLDDKKLLYRHSFCQTYAEFQHFSPEWKICLGVALWL
VAIGMGISIAMKAKLYGELPETFDDEHQSAQLRRIIQLQMNPITGISSKWCYHENKWK

>XP_017114377.1_Drosophila_elegans_COX4L
MSALRLIPRGNAGGNLSQIMTLSQMGRRLASGGDGIRLMVGEREVVGYGINGRPIYFDSQDC
PFPAIRYREVTPELCAIREKELGDWKKLSLDDKKRLYRHSFCQTFVEFQHFTPEWKICLGVAL
WLVALGLSISMVLKTKMYGQLPDTFDEDRRSAQLRRMIQLQMNPITGISSKWCYEENKWKSQ
LH

>XP_017073706.1_Drosophila_eugracilis_COX4L
MSALRLIPRFLPRNLGQMPIVGRRFASGGDGIRLMVGDREMVGYGINGRPIYFDTADCPFPAI
RFREVTPELCAIREKELDDWKSMSLDDKKQLYRHSFCQTFAEFQHVSPEWKVCVGTALWFLA
LGICISMILKAKMYPELPDTFSDDRQSAQLRRIIQLQMNPITGLSSKWCYHENKWK

>XP_016954589.1_Drosophila_biarmipes_COX4L
MSALRLIPRNLSQLATPTRMMIGRRFASGGDGIRLLIGDREVVGYGINGRPLYFDSQDCPFPAI
RYREVTPELCAVREKELGDWKKLSLDDKKLLYRHSFCQTYAEFQHFSPEWKICLGVALWLVA
LGMGITIVLKAKMYGELPETFDDVHQSAQLRRMIQLQMNPITGISSKWCYHENKWK

>XP_001966770.1_Drosophila_ananassae_COX4L

MSALRMLPRARPMHLDSSVVMGLRFASGDQTRIMSGDREVVGYGINGRPIYFDSQDCPFPAI

RFRELTPEVCAIREKELGDWKKLSLCEKKMLYRHSFCQTYAEFQKFTPDWKLVLGLGLWSLAI

GCAITVISKLNLYNDPPETFEEDRRSAQLRRIIQLQMNPITGLSSKWCYERNQWK

>XP_022228406.1_Drosophila_obscura_COX4L

MSIIRALSLHLHQQCHRILPQQWALLAMVRRETHHDGTRLMSGDREVVGYGINGNPIYIDCVE

FPFPAIRYREVTAELCAAREKELGDWNSLSLQEKKLLYRHSFCQTYAEFQHFTPDWKLAMGV

GFWSAAIGLLISLSYYFKLYGPVPETYAEDRRQAQLRRIIQLQMNPITGLSSKWCYHTNKWK

>XP_002064201.1_Drosophila_willistoni_COX4L

MLSLRPLSKLQTLRSSLCRSGGPAVVAAGSIQRQTHHDGTRLMSGDREVVGYGINGSPIYIDC

VEFPFPAIRYREVTPELCAVREKELGDWKNLSLEEKKSLYRHSFCQTYAEFQHFTPDWKLVLG

IGFWSIAIGIMMTILYNTKIYDPLPETYDEDRRQAQLRRIIQLQIQPITGISSKWCYHTNTWK

>XP_002019540.1_Drosophila_persimilis_COX4L

MSVMRALGLRLHNQFQQVLPQRGAVMAMMRRGTHHDGTRLMSGDREVVGYGVNGNPIYID

CVEFPFPAIRYREVTPELCAVREKELGDWKALSIQDKKLLYRHSFCQTYAEFQHFTPDWKLVI

GVGFWSAAIGLLISLSYYFKLYDPVPETYAEDRRQAQLRRIIQLQMNPITGLSSKWCYHTNKWK

>XP_002137971.2_Drosophila_pseudoobscura_COX4L

MSVMRALGLRLHNQFQQVLPQRGAVMAMMRRGTHHDGTRLMSGDREVVGYGVNGNPIYID
CVEFPFPAIRYREVTAELCAVREKELGDWKALSIQDKKLLYRHSFCQTYAEFQHFTPDWKLVI
GVGFWSAAIGLLISLSYYFKLYDPVPETYAEDRRQAQLRRIIQLQMNPITGLSSKWCYHTNKWK

>XP_002055279.1_Drosophila_virilis_COX4L
MNSLRCAGLRVSGAAAAAACRPSAGRMTMLPAVMRRLTHHDGTRCMSGEREYVGYGVNGN
PIYIDCADFPFPAIRYREVTPEICALREKELSDWKKLSLQEKKALYRHSFCQTYSEFQHFTPEW
KLVVGIGLWSIALSILLTLAVNLIYDELPETFDEERRQAQLRRIIGLQMQPITGLSSKWDYSQNK
WK

>XP_030379296.1_Scaptodrosophila_lebanonensis_COX4L
MSGEREVVGYGVNGSPIYIDCVEFPFPAIRYKEATPEICALREKELSDWKKLSLQEKKTLYRYS
FGQTYAEFQHFTPEWKLIVGIGLWACAIGFLISLSYCAKLYGPFPETFEEERRQAQLRRIIQLEM
QPITGLSSKWDYEKNKWK

>XP_017145095.1_Drosophila_miranda_COX4L
MSVMRALGLRLHNQFQQVLPQRGAVMAMMRRGTHHDGTRLMSGDREVVGYGVNGNPIYID
CVEFPFPAIRYREVTAELCAVREKELGDWKKLSLKDKKLLYRHSFCQTYAEFQHFTPDWKLVI
GVGCWSVAIGLLLSLSYYFKLYDPVPETFAEDRRQAQLRRIIQLQMNPITGLSSKWCYHTNKW
K

>XP_023292338.1_Lucilia_cuprina_COX4L

MALQICKLISRQQLWKSFKSPPLTISKRATSHDYTNTMCGKREYVGFGVNGAPIYVDLVDFPM
PAIRFQEPSPEICALRKKEEDDWKKLSKDEIKRLYRYSFCRTFAEMKAPTGEWKLHLGIALWA
CTIGLLFCYTVNVYHQDLPDTFAEDRRQAQLKRIIALEMNPITGLASKWDYEVGDWK

>XP_014094089.1_Bactrocera_oleae_COX4L
MQSARRPLVRLAGRFGIRLTHSDQVMERIGKREIVGYGWNGSPCYHDRLDYPMPAVRFREP
DPEICALREKETGDWRKLSIDEKKQLYRYSFRKTFAEMKAPTSDWKFSLGVALIAVSIGIWLSQ
SYAHGIYPEYPETFEEKRRSAQLKRMIALEVNPVTGLASKWDYEKDRWK

>XP_011189219.1_Zeugodacus_cucurbitae_COX4L
MQSPQNPFVRLARRFGIRLTICRQTHSDQVMERVGKREIVGYGWNGSPCYSDRLDYPMPAV
RFREPDTEICALREKETADWRKLSMDEKKQLYRYSFCKTFAEMKAPTSDWKFSLGAALIAVSI
GIWLSQSYFHGIYPEYPETFEEKRRSAQLKRMIALEVNPVTGLASKWDYEKDRWK

>XP_005188663.1_Musca_domestica_COX4L
MALQIEKLIARQGLIKYLNHFPRLAPQKRYTSFDFTNTKCGKREYVGFGVNGAPIYCDVAEFPM
PAIRFREPDATICALREKEKGDWKKLSKEDIKTLYRYSFCQTFAEFKAPTGEWKHHLGVGLWA
CAIGVVWTAFVNWYHRDLPDTFDEDRRQAQLKRIIALEMNPIEGISSQWDYEVGDWK
